# The Genome of the Trinidadian Guppy, *Poecilia reticulata*, and Variation in the Guanapo Population

**DOI:** 10.1371/journal.pone.0169087

**Published:** 2016-12-29

**Authors:** Axel Künstner, Margarete Hoffmann, Bonnie A. Fraser, Verena A. Kottler, Eshita Sharma, Detlef Weigel, Christine Dreyer

**Affiliations:** 1 Department of Molecular Biology, Max Planck Institute for Developmental Biology, Tübingen, Germany; 2 Guest Group Evolutionary Genomics, Max Planck Institute for Evolutionary Biology, Plön, Germany; Xiamen University, CHINA

## Abstract

For over a century, the live bearing guppy, *Poecilia reticulata*, has been used to study sexual selection as well as local adaptation. Natural guppy populations differ in many traits that are of intuitively adaptive significance such as ornamentation, age at maturity, brood size and body shape. Water depth, light supply, food resources and predation regime shape these traits, and barrier waterfalls often separate contrasting environments in the same river. We have assembled and annotated the genome of an inbred single female from a high-predation site in the Guanapo drainage. The final assembly comprises 731.6 Mb with a scaffold N50 of 5.3 MB. Scaffolds were mapped to linkage groups, placing 95% of the genome assembly on the 22 autosomes and the X-chromosome. To investigate genetic variation in the population used for the genome assembly, we sequenced 10 wild caught male individuals. The identified 5 million SNPs correspond to an average nucleotide diversity (*π*) of 0.0025. The genome assembly and SNP map provide a rich resource for investigating adaptation to different predation regimes. In addition, comparisons with the genomes of other Poeciliid species, which differ greatly in mechanisms of sex determination and maternal resource allocation, as well as comparisons to other teleost genera can begin to reveal how live bearing evolved in teleost fish.

## Introduction

The advent of cost-effective and high-throughput sequencing technologies has brought genomics into the field of evolutionary biology. In combination with progress in computational biology, next generation sequencing enables researchers to study closely related populations or species using full genome sequencing (e.g. [1], [2]), to investigate how genomic variation is structured among and within species, and to determine how genomic variation relates to phenotypic and environmental variation (e.g. [3–5]). With this information we can now address fundamental questions in evolutionary biology, such as how populations adapt to new or changing environments, whether traits are caused by few genes of large effect or many genes of small effect, and what the relative importance of demography and selection are in shaping variation.

The first crucial step required to address these questions is the construction of a high-quality reference genome. While, many ‘reference-free’ methods for studying genetic variation exist (e.g. *de novo* RAD-seq, *de novo* transcriptome assembly [6, 7]), they have substantial inherent shortcomings, such as limited information about linkage among genes, ortholog-paralog ambiguity, difficulties in differentiating gene loss from insufficient sampling, and mis-annotation [8]. Some of these issues will likely be particularly troublesome in teleost fishes because a whole genome duplication event occurred early in diversification of teleosts, and various lineages have since independently undergone additional genome duplications.

In addition to the basal whole genome duplication being followed by rediploidization, which in turn leads to the loss of extra copies, functional diversification and neo-functionalization of paralogous gene copies are prominent features of genome evolution in teleosts [9, 10] (reviewed in [11]). For this reason, and because of the gigantic number of diverse species in this class, teleost fishes provide a rich resource for studying the evolution of molecular function of genes and whole genome organization. Consequently, a contiguous, annotated reference genome assembly is yet another milestone in fish evolutionary biology [12].

**Fig 1:**
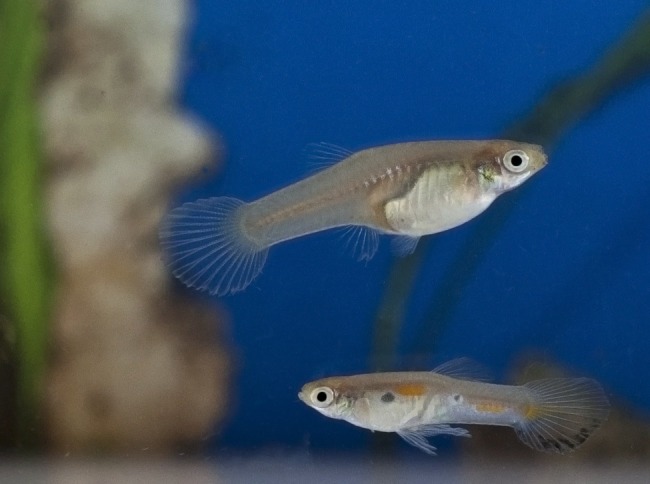
Guppy reference genome strain. Female (top) and male (bottom) from the inbred Guanapo strain.

Here we focus on the Trinidadian guppy (*Poecilia reticulata*) as a premier vertebrate model for the study of natural variation and local adaptation. The Trinidadian guppy is a small, live bearing freshwater fish with marked phenotypic dimorphism between the sexes and an XX/XY sex-determination system. In contrast to the inconspicuous reticulate pattern of the larger females, the complex patterns of adult males vary greatly within and between different natural populations (Fig 1). Not surprisingly, the guppy was one of the first vertebrate species for which sex-linked inheritance of traits could be demonstrated [13].

Comparative studies have demonstrated that guppies have convergently evolved similar adaptations to life with or without predators in the different rivers that drain the slopes of the Northern Range Mountains in Trinidad. These adaptations include male coloration, mating and schooling behavior, and life history traits [14, 15]. Population genetic studies have shown that natural low predation populations derive from independent colonization events by ancestral down-stream and high predation populations within each river drainage [16, 17]. The predation regime is regarded as a major driving force for adaptation. Some predators in downstream localities prey predominantly on large, mature guppies, favoring the evolution of male guppies with less coloration and covert mating tactics. Barriers exclude these predators from upstream localities. The few predators found there, predominantly the killifish *Rivulus hartii*, eat fewer guppies and tend to prey on smaller, immature fish. Under these conditions, sexual selection in the form of female preferences prevails over natural selection by predators, and males evolve to be more brightly colored [14, 15, 18]. When guppies are transplanted from high to low predation localities, measurable character shifts occur within three to ten generations [19, 20], indicative of selection from standing natural variation in the founder populations [14, 18, 21, 22]. Standing genetic variation provides a rich repertoire of alleles that allows for selection on beneficial alleles that enhance fitness in a changing environment. Owing to the maintenance of ancient genetic variation or current gene flow, beneficial alleles have already passed a ‘selection filter’ [23]. Furthermore, adaptive alleles may be maintained at high frequencies due to balancing selection. Negative frequency dependent selection has been shown to be operating in guppies, where males with rare color patterns have a higher probability of surviving [24] and a higher mating advantage [25].

The guppy has a haploid complement of 23 chromosomes, with an XX/XY sex-determination system. Early in the 20th century, Winge used crosses among inbred lines of guppies to show that many male color patterns are Y linked [13, 26]. Later investigators used quantitative genetic approaches to show that sires have much larger effects than dams on the inheritance of male size and coloration [27–29] which again argued for these traits being controlled in part by Y-linked genes. More recently, Tripathi and colleagues [30] used a classic F_2_ quantitative genetic framework with approximately 800 markers and 2,000 individuals for QTL mapping of size and coloration traits. They found several regions associated with both male size and color to be linked to the sex-determining locus, but also detected a number of additional QTL on different autosomes. Models of sex chromosome evolution predict that genes that benefit only the heterogametic sex, such as genes for conspicuous color patterns, will have increased evolutionary fitness if physically linked to the sex-determining locus through suppression of recombination [31]. Investigation of sex chromosome structure from various guppy populations and synaptonemal complex measurements have revealed polymorphisms between the X and Y chromosomes [32]. Guppy sex chromosomes are considered relatively young because the X and Y chromosomes are in most populations not morphologically distinct, in spite of there being many genes apparently linked to the non-recombining portion of the Y chromosome [33–35]. Consequently extended pseudoautosomal regions continue to be exchanged between X and Y [33, 35].

Both the evolution of sex determination in this species, and the genetic basis of local adaptation would greatly benefit from a high-quality genome assembly. We have therefore sequenced and assembled a female reference genome, using a combination of paired-end, mate-pair, and fosmid libraries. The vast majority of assembly scaffolds was then placed by genetic linkage on the 22 autosomes and the X-chromosome. Additionally, we describe genetic variation found in the reference genome’s source population, a high-predation site from the Guanapo River in North West Trinidad.

## Material and Methods

### Genome assembly and annotation

#### Founder fish sampling and fish housing

Founder fish for inbreeding were first-generation lab-reared guppies from the Lower Guanapo River (Twin Bridge North West Trinidad, PS 91100 77800) where they are neither endangered nor protected. The specimens were kindly donated by Dr. David Reznick, UC Riverside, in 2009. Fish collection and export of these fish was approved by the Ministry of Agriculture, Land and Marine Resources, Republic of Trinidad and Tobago, conforming to their legislation. Since their collection progeny was kept and bred at the Max Planck Institute for Developmental Biology, Tübingen, according to German legislation. The facility was approved by the Regierungspräsidium Tübingen, registration number 35/9185.46. Fish required for preparation of DNA were anesthetized with a lethal dose of MS222 before being stored in 95% ethanol.

#### Genome sequencing

DNA from a 5^th^-generation female was used to prepare Illumina paired-end libraries with insert sizes of 240 to 460 bp and DNA from female offspring of the same lineage at generations 6 to 8 (six individuals total) was used to construct Roche/Illumina hybrid mate-pair libraries of 3 to 20 kb length; for details see [36]. Briefly, mate-pair libraries were prepared by ligating a circularization adaptor (Roche) and fragments were selected. The fragments were then circularized by Cre Recombinase, following the Roche protocol for 454 sequencing. Linear DNA was digested before circularized DNA was fragmented. Illumina paired-end standard adaptors were ligated onto these fragments, mate-pairs (of 180 to 480 bp length) were amplified and sequenced from both ends on the Illumina GA II platform. Long-jump 40 kb fosmid libraries were constructed from a single female offspring at generation 8 using the Nx 40 kb mate-pair cloning kit from Lucigen (Middleton, USA). Fosmid clones were amplified, fosmid DNA extracted in bulk, digested with *BfaI* and end fragments of 8–9 kb size (vector including insert ends) were selected. After recircularization, and digestion of linear DNA, mate-pairs were amplified by PCR and sequenced on the Illumina HiSeq2000 platform. See S1 Table for details about library sizes and sequencing yield.

#### Read filtering and quality trimming

All genomic libraries were retrieved and converted to FASTQ format using the *import* and *convert* commands in shore version 0.7.1 [37]. To remove PCR duplicates for a non-random fragment representation, each library was scanned with the *filterPCRdupl*.*pl* script (Version 1.01) included in ConDeTri version 2.0 [38]. For paired-end libraries, the first 50 bp of both reads of a pair were compared, and for mate-pair libraries the first 35 bp.

Mate-pair libraries were screened for the following adapter sequences:

5’–TCGTATAACTTCGTATAATGTATGCTATACGAAGTTATTACG– 3’

5’–CGTAATAACTTCGTATAGCATACATTATACGAAGTTATACGA– 3’

Screening was conducted using cutadapt version 1.1 [39], with an error rate of 0.15, an overlap of 6 bp, minimum read length of 35 bp, and matching wildcards. The screening resulted in two sets of mate-pairs: one set that contained parts of the adapter sequences and the other set without any adapter sequence. All other libraries were filtered for low quality bases using ConDeTri version 2.0 (using default parameters for paired-end libraries and with parameters–rmN–hq 20 –minLen 25 for mate-pair libraries).

#### Estimating mate-pair library insert sizes

To make use of mate-pair library information even in the absence of adapter sequences in the reads we first prepared a draft assembly. For this assembly SOAPdenovo version 1.05 [40] (kmer size 27, -d 1, -D 2, -F) was used. In detail, all paired-end libraries were used for contig building, but only mate-pair libraries with adapter sequence were included; these mate-pair reads were added stepwise according to their insert size (smallest to longest) for eight rounds of scaffolding. Mate-pair reads without adapter sequence were mapped to the assembly using bwa version 0.6.1 [41] with default parameters. Mate-pairs that mapped within 1 kb distance of each other were excluded from further assembly steps to prevent possible paired-end contamination in the data. Insert sizes for the remaining reads were estimated per library based on distances between the reads.

#### Fosmid 40 kb insert library

The fosmid 40 kb library was treated slightly differently from the mate-pair libraries. First, *phiX* sequences were removed by mapping the library to the *phiX* genome sequence using bwa with default parameters. Then, reads were filtered for PCR duplicates and low quality bases, similar to the mate-pair libraries. Finally, reads were trimmed from the 3’ end to keep 50 bp per read due to the low quality towards the 3’ end of the reads. Insert sizes of the filtered and trimmed reads were estimated as described above (using the SOAPdenovo assembly).

#### Genome assembly

*T*he final genome assembly was built with Allpaths-lg version 43668 [42] using default parameters. Due to a quality correction step in the Allpaths-lg pipeline, we used PCR filtered libraries for the paired-end and mate-pair libraries, and PCR-filtered and quality-trimmed fosmid libraries. Overlapping paired-end libraries were used for contig building (insert sizes of 240 and 270 bp, library ID 1–5). Longer insert paired-end libraries (insert size 460 bp, library ID 6–8), mate-pair libraries (ID 9–19), and the fosmid library (ID 20) were incorporated in the scaffold building process. Allpaths-lg was used to estimate the heterozygosity rate of the reference genome sample.

After assembly, all paired-end reads were mapped back to the genome assembly using bwa version 0.6.2 to estimate the proportion of the genome that was not covered by paired-end reads. Only a very small proportion of the genome assembly was not covered by paired-end reads (224,382 bp or 0.03%). Additionally, the assembly was screened for regions of runs of Ns. These regions occur during the scaffolding process and denote that sequence information between two contigs might be missing.

#### Contamination removal

To exclude cross contamination in the assembly from other organisms, we aligned the final genome assembly against the NCBI *nt* database (blastn version 2.2.21, e-value cutoff 10^−5^ [43]), reporting the best hit only. Scaffolds with hits only against non-vertebrate organisms were excluded from the assembly. This strategy excluded eight scaffolds with combined length of 27,088 bp (0.004% of the assembly) from the final assembly. Additionally, a more stringent contamination screen for adaptors was performed by NCBI resulting in 1,260 bp of potential adaptor sequences that were removed from the assembly. Visual inspection did not reveal any signs of mis-assemblies in these regions.

#### Genetics map integration

Scaffolds were anchored on linkage groups using a genetic linkage map built from 5,493 RAD-seq markers (for details about the linkage map see S1 File). Markers were aligned against the assembled genome (blastn version 2.2.27+, e-value < 10^−20^ [44]) and only markers with unique hits were used to anchor scaffolds using the method described in [1]. Adjacent scaffolds were separated by a character string of 100 Ns. Scaffolds that could not be reliably anchored to one of the linkage groups were grouped into LG Un.

#### Assembly validation

Guppy expressed sequence tags (ESTs), were downloaded from the NCBI EST database (accessed 2013-09-25) and blasted (blastn version 2.2.27+, e-value < 10^−10^ [44]) against the draft genome assembly. Additionally, a set of 454 transcriptome sequences [45] was downloaded (http://www.bio.fsu.edu/kahughes/Databases.html) and blasted (blastn, e-value < 10^−10^) against the draft genome sequence. See S1 File for further assembly validation steps.

#### Repeat content

A guppy specific repeat library was built using the draft guppy genome assembly and RepeatModeler version open-1.0.5 [46] in combination with ab-blast version 2.2.6 [47] (default parameters used for both programs). The resulting repeat-library was applied to identify and mask repeats in the draft assembly using RepeatMasker version open-3.3.0 [48], with ab-blast used as the search engine (default parameters).

#### Gene set annotation

Genes were annotated using ‘The NCBI Eukaryotic Genome Annotation Pipeline’ (http://www.ncbi.nlm.nih.gov/genome/annotation_euk/process/, accessed 2014-09-05). This automated pipeline annotated genes, transcripts and proteins on the draft genome assembly (NCBI Poecilia reticulata Annotation Release 100).

#### Genes with potential functions in pigment pattern development, vision, growth and sex differentiation

To identify protein coding genes with putative functions in pigment pattern development, vision, growth and sex differentiation, reciprocal blast searches were performed using a sample of 624 published coding sequences (S13 Table), mostly from related fish species. Resulting high-scoring segment pairs (HSPs) were manually scrutinized for e-value (< 10^−20^), percent identity (>90%), length, and reading frame. Further, predicted gene models from scaffolds localized on LG12 were searched against NCBI *nt* and *nr* databases as well as Ensembl (version 71) databases of medaka, stickleback and platyfish. To resolve positions of LWS-1 (A180), LWS-2 (P180 and LWS-3 (S180) in the cluster on scaffold 43, all exons were scrutinized for best e-value (≤ 10^−40^), percent identity and coding strand. Further, we aligned a Cumaná genomic BAC (GenBank HM540108.1) to genomic scaffold 43. A region of 32,500 bp length in the reconstructed BAC sequence corresponds to the opsin gene cluster that was aligned to about 38,300 bp on scaffold 43 of our assembly (96 to 98% identity, excluding few gaps). Reciprocal blast alignment revealed stretches of N in the Allpaths-lg assembly (up to 4,500 bp) as the main reason for length discrepancy. We inspected this region by eye but did not find evidence that points towards a mis-assembly in this region. A potential explanation for the length discrepancy is wrongly estimated insert sizes of the mate pair libraries for this particular region. Another possible explanation is a length difference for this particular region between the Cumaná and Guanapo strains.

#### Small non-coding RNAs and transfer RNAs (tRNAs)

Small non-coding RNA loci were annotated using Infernal version 1.1rc1 [49] (e-value threshold 10^−4^) in combination with Infernal Rfam database version 1.1. To annotate tRNAs we additionally ran tRNA-Scan version 1.3.1 [50].

### Alignments

#### Pairwise alignments/Synteny analysis

The guppy genome was aligned to repeat-masked versions of the medaka (*Oryzias latipes*) and stickleback (*Gasterosteus aculeatus*) genomes (Ensembl version 71) using NUCmer version 3.1 from the MUMmer package version 3.23 [51]. Alignments were visualized with Circos plot version 0.67–7 [52].

#### Three-way alignments

Coding sequence annotations for the guppy were downloaded from GenBank (Accession GCF_000633615.1) and coding sequences from platy (*Xiphophorus maculatus*) and medaka (*Oryzias latipes*) were downloaded from Biomart (Ensembl 70). Three-way 1:1:1 orthology sets were identified using ProteinOrtho version 5.11 (parameter settings: minimum similarity for additional hits 0.8, blastp+). In total, 10,840 1:1:1 orthologs were identified. Next, codon sequences were aligned using prank version 140603 [53] with an empirical codon model.

### Molecular evolution analyses

#### Substitution rate estimates

Substitution rates were estimated separately for synonymous (*d*_*S*_*)* and nonsynonymous (*d*_*N*_) substitutions per nucleotide using a maximum likelihood method, implemented in the Codeml program (model = 1, star-like user tree specified according to the phylogeny) of the Paml package v4 [54]. Alignments with *d*_*S*_ > 2 along any branch were excluded to minimize statistical artifacts from short sequences and saturation effects in *d*_*S*_ (no alignment showed an estimated *d*_*N*_ > 2). The final data set comprised 9,111 1:1:1 orthologs with mean *d*_*S*_ estimates of 0.052 (±0.033 s.d.), 0.058 (±0.040 s.d.), and 0.949 (±0.262 s.d.) for the platy, guppy, and medaka branches, respectively. Estimates for average *d*_*N*_ were 0.008 (±0.010 s.d.), 0.008 (±0.013 s.d.) and 0.093 (±0.066 s.d.) for the platy, guppy, and medaka branches, respectively.

### Mutation rate estimates

#### Parent-offspring trios were used

We crossed a Quare female (laboratory strain, originally from the Quare River in North East Trinidad) with an EnUlmBL male (laboratory strain, presumably originally from Venezuela). RAD-seq libraries for the parents and five F_1_ individuals were prepared using the restriction enzymes *PstI* and *MseI* and 8 unique barcodes for each parent and one unique barcode for each F_1_ individual [55]. The RAD-seq libraries, with an approximate insert size of 120–220 bp, were sequenced single-end on an Illumina HiSeq 2000 lane.

The raw reads were obtained from the sequencing platform, converted to FASTQ format and de-multiplexed using shore version 0.8.1. Read mapping was performed separately for each individual with bowtie2 version 2.1.0 [56]. Mapping results were enhanced by local realignment using GATK version 2.4–9 [57]. Single nucleotide polymorphism (SNP) detection was performed using GATK UnifiedGenotyper (default parameters). High quality bases were extracted using the *mpileup* command, implemented in SAMtools version 0.1.18 [58] (BAQ score cutoff 20).

To detect *de novo* mutations, SNP calls from the parents were compared with the offspring using only sites that were covered by at least 10 reads. Approximately 16 million bases reached the base quality cutoff and between 0 and 2 new mutations were detected per F_1_ individual. None of the *de novo* mutations were detected in more than one individual.

### Resequencing

#### Sampling and sequencing

Ten males from a downstream high-predation population were collected in 2011 (PS 91100 77800, Twin Bridges) from the Guanapo drainage in North West Trinidad. Fish were euthanized with MS222 and stored in 95% ethanol.

Paired-end DNA sequencing libraries were prepared according to the "Illumina Paired End Preparation protocol"; using unique barcoded Illumina TruSeq adaptors for each individual. The PCR amplified fragments were size selected on a 2% Low Range Ultra Agarose (Bio-Rad) gel. Libraries were pooled and sequenced on two flowcell lanes with an Illumina HiSeq 2000 instrument, aiming for approximately 10x coverage per individual (101 bp read length).

#### Data preparation

Libraries were checked out from the sequencing platform using the shore *import* command version 0.8.1 to retrieve raw data. Raw reads were converted to fastq files using the shore *convert* command (see S2 Table for details about sequencing yield per sample).

Paired-end reads were mapped to the reference genome using bowtie2 version 2.10, applying the ‘end-to-end’ mapping option (no read-clipping) in the ‘very-sensitive’ mode. Discordant alignments for paired reads were suppressed. Mapping was enhanced for each individual by local realignment as implemented in GATK version 2.4–9 (RealignerTargetCreator, IndelRealigner) and duplicates were marked using Picard version 1.89 (http://picard.sourceforge.net, last accessed 2014-07-09).

#### SNP calling

SNPs were called using three different variant calling programs: GATK UnifiedGenotyper, freebayes version 0.9.9 [59], and SAMtools mpileup version 0.1.18. GATK UnifiedGenotyper and SAMtools mpileup were run with standard parameters, freebayes was run with ‘—no-indels—no-mnps—no-complex—use-mapping-quality’ parameters. SNPs called by all three SNP calling approaches (~0.7 million) were selected as input data for base quality recalibration with the default set of covariants (GATK BaseRecalibrator). Base quality of the mapping files was adjusted using GATK PrintReads and the resulting bam files were then used as input for a second round of SNP calling with GATK UnifiedGenotyper (standard parameters). Variants with a quality by depth annotation above 10.0 (QD>10.0) were selected as the training set for quality recalibration and filtering using GATK (~2.1 million). Next, we conducted a third round of variant calling using GATK UnifiedGenotyper. As there was no significant difference in the number (and location) of SNPs between the second and the third round, the third round SNPs were used as the final variant set for downstream analyses. In the final SNP set, the average transition-to-transversion ratio was 1.35. The predicted effect of each SNP was annotated using SnpEff version 3.3h [60].

#### Pairwise nucleotide diversity estimation

The genome was divided into non-overlapping windows of 50 kb for each scaffold separately. The last window of a scaffold was disregarded if it was shorter than 50 kb. Scaffolds were ordered and oriented along chromosomes as described above (see section ‘Physical assembly’). Windows with at least 25 kb coverage of unique sequences were retained for downstream analyses; unique sequences were defined as the sites within each window that were neither N nor repeat masked. Additionally, we required that these windows were covered by sequence information from at least 7 individuals (i.e., a missing data threshold of 30%). For each retained window, average pairwise nucleotide diversity (π, 0…1) was computed for non-repetitive sites using compute, as implemented in the analysis package version 0.8.3 (https://github.com/molpopgen/analysis, last accessed 2014-11-11) found in the libsequence library [61] with default parameters.

#### Demographic inference

To infer the effective population sizes (*N*_*e*_) history of the Guanapo high predation population, a coalescent-based hidden Markov model (PSMC) was applied as described in [62]. This method infers ancestral *N*_*e*_ over time, exploiting a probabilistic model of coalescence that accounts for recombination and changes in heterozygosity rates along a single diploid genome. We ran PSMC with standard parameters for each individual using recalibrated bam files (see above for details) on long, repeat masked scaffolds (size >10 kb). To visualize the results, *psmc_plot*.*pl* was used, assuming a mutation rate of 4.89x10^-8^ bp per generation and a generation time of 0.5 years [20]. Results were plotted for 800 to 25,000 generations ago, which translates to 400 to 12,500 years before present. The lower bound was set because estimates more recent than 800 generations are difficult to predict by the PSMC method [62].

### Male-specific sequences

#### Resequencing and assembly

From each of the 10 males used for resequencing, reads that did not align to the female genome were extracted using the view command from the SAMtools package. Reads from all individuals were pooled together, resulting in 35 million read pairs. To lower the computational burden, ten million read pairs were randomly extracted from this pooled set of unmapped reads and assembled using Trinity version r20140717 [6]. Contigs shorter than 1,000 bp were discarded and only the longest isoform was kept for each assembled component. Other assemblers (SOAPdenovo, Velvet [63] and ABySS [64]) were tested for the male-specific assembly as well, but resulted in higher fragmentation compared to the Trinity assembly (data not shown).

#### Annotation

We used blastn to compare contigs against *env-nt* and *nt* databases (blastn version 2.2.30+, e-value < 10^−5^, database version 20141104) to screen for contamination. Contigs with hits against *env-nt* longer than 5% of the contig size were removed. Additionally, contigs with no hits against *nt* were removed. The remaining contigs were blasted against the female genome (blastn, e-value < 10^−10^) and all contigs with alignment length longer than one-quarter of the contig length were discarded. The resulting contigs were compared to the *nr* database (blastx, e-value < 10^−10^, database version 20151112). Only hits with at least 120 amino acids of target protein coverage, 50% identity and an e-value < 10^−40^ were kept.

### Statistical analysis

If not stated differently, all statistical tests were performed using R version 3.1.1 [65]. Where necessary, we used Bonferroni correction to adjust significance thresholds for multiple testing.

### Data deposition

All short read data generated from this project have been made available via the NCBI Short Read Archive (NCBI SRA study accession SRP038017). Accession numbers for genomic libraries can be found in S1 Table, and for population samples in S2 Table. Accession numbers for the samples used to estimate mutation rates are SRR1503964 and SRR1503965 for the parents (female, male), and SRR1503967-SRR1503971 for the five F_1_.

The genome assembly was submitted to NCBI under BioProject PRJNA238429. The entire project has been deposited at DDBJ/EMBL/GenBank under the accession AZHG00000000. The version described in this paper is version AZHG01000000. The assembly comprises linkage groups 1 to 23, all unplaced sequences, and the mitochondrial genome.

The scaffold accessions appear in the WGS_SCFLD line at the bottom of the WGS master record, AZHG00000000 (CM002706-CM002728 = chromosomes, KK214999-KK218026 = scaffolds).

## Results

### Reference genome assembly

In order to generate a reference genome for the guppy, we selected a single female and her female descendants from a high-predation population in the Guanapo drainage. This lineage had been inbred by brother-sister matings in the laboratory for five generations to reduce heterozygosity in the genome. We estimated heterozygosity to be about 1 SNP per 400 bp in the individual used for paired end genome sequencing. We used offspring of this female from later generations to produce a range of Illumina libraries, with insert sizes of up to 40 kb, and generated approximately 225 Gb of raw data (see S1 Table for additional information about libraries and insert sizes). After removing PCR duplicates about 148 Gb of sequence data remained, which we assembled into 3,028 scaffolds of a total length of 732 Mb. The longest scaffold (scaffold 0) is over 21 Mb long. Half of the assembly is represented by 43 scaffolds that are at least 5.3 Mb long (N50), and 90% of the assembly by 163 scaffolds larger than 1 Mb (N90; see Table 1 for further assembly details). The assembly size is within previous estimates of 740 to 900 Mb for the guppy genome [66], with 2*n* = 46 chromosomes [32, 34]. A *k-mer* frequency approach estimated a genome size of 779.8 Mb for the female genome (S1 File), which is just slightly larger than the assembled genome.

**Table 1:** Overview of assembly and annotation for the female guppy genome.

Contigs longer than 1kb	44,571
Total length of all contigs	663,389,323 bp
N50 length of contigs	35,577 bp
Scaffolds	3,028
Total length of all scaffolds	731,579,643 bp
Length of unclosed gaps	66,967,969 bp
Median size of gaps in scaffolds	535 bp
Lengths of scaffolds anchored on linkage groups	696,674,853 bp
Scaffolds anchored on linkage groups	284
Longest scaffold	21,430,553 bp
N50 length of scaffolds	5,270,359 bp
N90 length of scaffolds	1,021,883 bp
Scaffolds longer than N50 length	43
Scaffolds longer than N90 length	163
GC content	39.3%
Protein-coding genes	22,982
Pseudogenes	249
Fraction of transposable elements	21.3%

To estimate the completeness of the assembly, we mapped expressed sequence tags (ESTs) [65] and a Roche 454 transcriptome [45] from other guppy strains to our newly constructed reference. The majority of both ESTs (15,579/16,220; 96.0%) and 454 contigs (50,188/54,981; 91.28%) could be located on the genome assembly.

Visual pigment genes (opsins) have been extensively characterized in guppies [67–71]; we therefore searched our genome assembly for these loci as a further validation of the assembly’s completeness. Using published opsin cDNA and genomic sequences from guppies [67, 68, 70] and from closely related poeciliid species [69], we confirmed the presence of rhodopsin and nine cone opsin genes (S3 Table). Seven are in two clusters on LG5, one including the green-sensitive RH2-1 and RH2-2, and the other including the blue sensitive SWS2A and SWS2B and three red/orange-sensitive LWS genes. A fourth, retrotransposed LWS-4 gene is on LG2, and the gene encoding the UV-sensitive SWS-1 is on an unplaced scaffold (LG Un). These results confirmed the high quality of our assembly.

Based on comparing RAD-seq tags in parents and their F_1_ offspring, we calculated a mutation rate of 4.9 x 10^−8^ bp^-1^ generation^-1^. This is in a similar range as for Midas cichlids, which have an estimated mutation rate of 6.6 x 10^−8^ bp^-1^ generation^-1^ [72]. Our assembly should thus also be useful for detecting *de novo* mutations that may contribute to local adaptation in addition to standing variation.

### Genome annotation

A total of 22,982 protein-coding genes and 249 pseudogenes were predicted. Additionally, we annotated 439 tRNA genes for the 20 standard amino acids, 707 microRNA loci, and 160 snoRNA loci (S4 Table).

**Fig 2:**
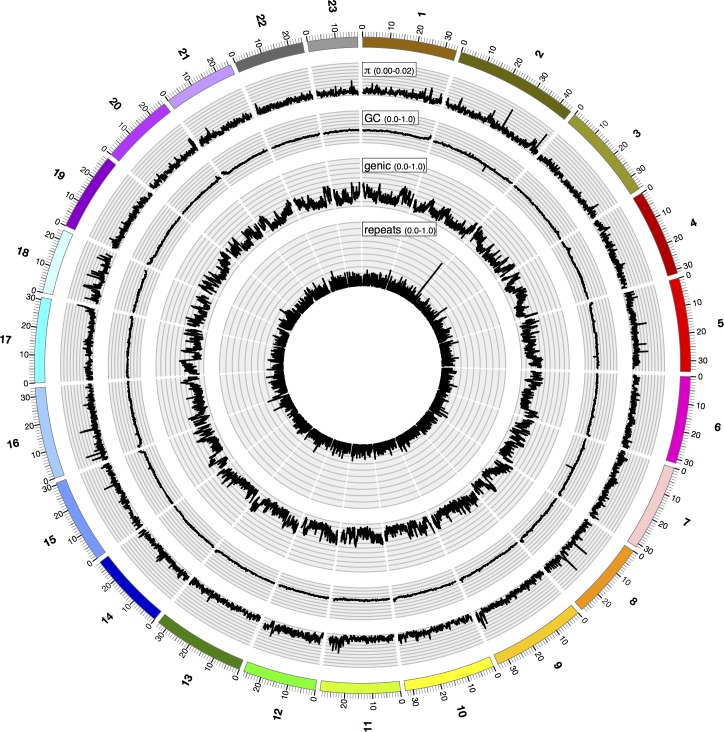
Sequence characteristics for each linkage group. Linkage groups are indicated on the outside. Small numbers indicate distances along each linkage group in Mb. Estimates for nucleotide diversity, π, GC content, and genic (exons and introns) and repeat density are averaged in 50 kb windows. Note that repeats can be located in genic regions.

Repetitive sequences made up approximately 20% (156 Mb) of the assembly (see S5 Table for further details). Given the difficulty of assembling highly repetitive centromeric regions, these may be underrepresented. The average GC content of the genome is 39.3%, without clear signs of isochore organization along the 23 chromosomes (Fig 2), though some chromosomes show slightly elevated GC content towards the ends of the linkage groups.

### Synteny with other fish genomes

**Fig 3:**
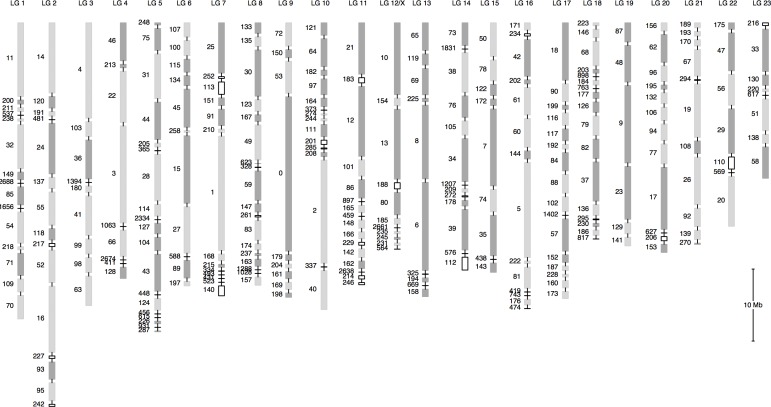
Distribution of anchored scaffolds along linkage groups. Grey outlined boxes denote scaffolds with at least two markers. Scaffolds in the forward orientation are solid grey and in the reverse orientation are dashed light grey. Black outlined boxes and horizontal black bars denote scaffolds with just one marker and unknown orientation. Spacing between scaffolds was set arbitrarily to 500 kb.

**Fig 4:**
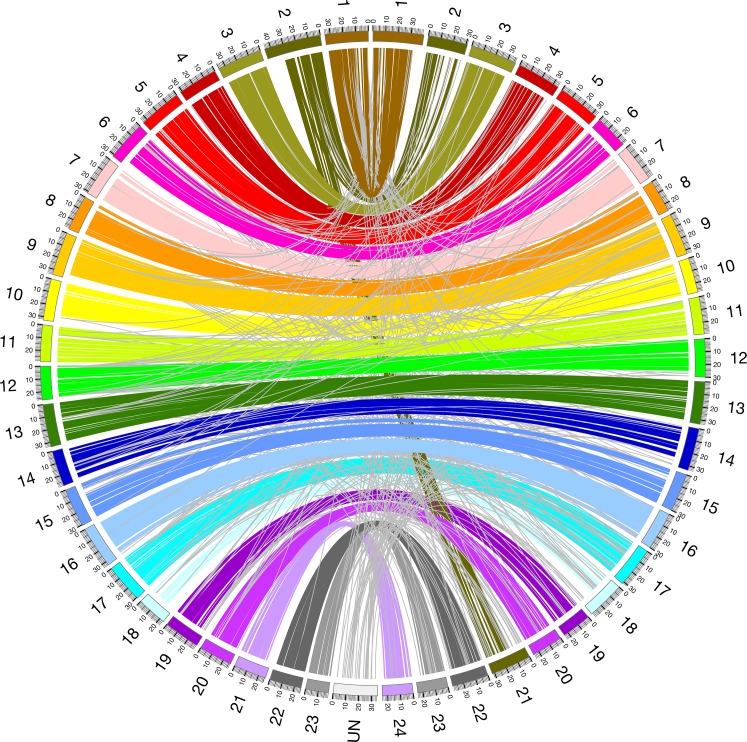
Whole genome alignment between Guppy and Medaka. Circos plot of syntenic relationship between guppy (left) and medaka (right) chromosomes. Minimum block length 500 bp. Light grey lines indicate non-syntenic alignment blocks or blocks not assigned to any guppy linkage group (UN).

Using a high-density linkage map comprising 5,493 markers, more than 95% of the assembly could be anchored to 23 linkage groups (LGs), which corresponds to the guppy’s haploid set of 23 chromosomes. Estimated chromosome sizes range from 18 and 46 Mb (Figs 2 and 3, S6 Table). The longest chromosome, LG2, is the product of a fusion between ancestral chromosomes that correspond to chromosomes 2 and 21 of medaka (*Oryzias latipes*), and groups II and XVI of stickleback (*Gasterosteus aculeatus*) (Fig 4, S1 Fig). Almost all linked scaffolds could be oriented, as they contained at least two genetic markers with recombination events between them (Fig 3). An alignment of the guppy and medaka genomes confirmed extensive synteny (Fig 4), as had been previously deduced from mapping homologs of guppy genetic markers to the medaka genome [30].

### Sex chromosomes

**Fig 5:**
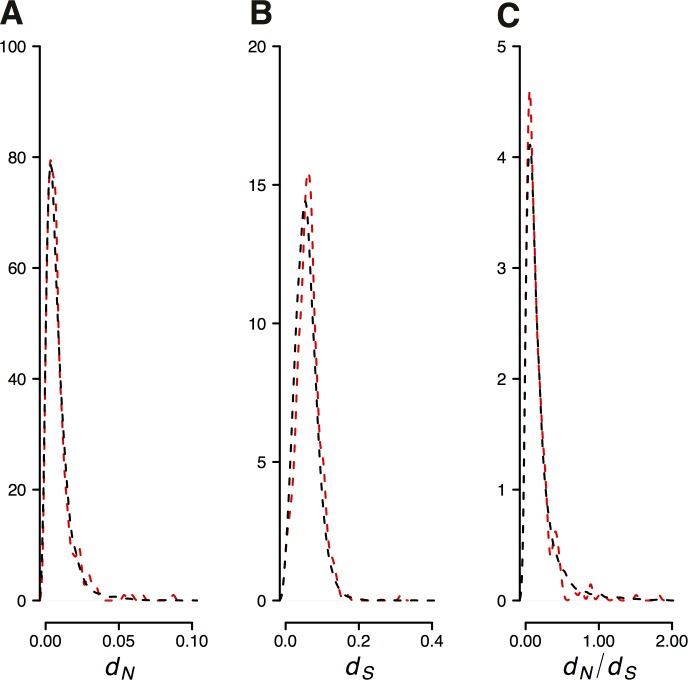
Evolutionary rates along X-chromosome (LG12) and autosomes. Density plots (A) of the rates of nonsynonymous (*d*_*N*_), (B) synonymous substitutions per nucleotide (*d*_*S*_) and (C) the ratios of *d*_*N*_ and *d*_*S*_ (*d*_*N*_*/d*_*S*_) between LG12 (red dashed lines) and autosomes (black dashed lines).

LG 12 corresponds to the X-chromosome, and the genetic map allowed us to assign 26.4 Mb of assembled sequence to this chromosome (Fig 3, S6 Table). Full genome alignments revealed synteny of guppy LG12 to chromosome 12 of medaka and group XIV of stickleback (S1B and S1C Fig). Gene and repeat content do not significantly differ between the X-chromosome and autosomes (Table 2) and overall GC content is very similar as well (39.0% *vs*. 39.4%). Rates of protein coding evolution, as estimated from the ratio of nonsynonymous to synonymous substitutions, *d*_*N*_*/d*_*S*,_ are not significantly different in the guppy branch between the X-chromosome and autosomes (Mann-Whitney *U* test, p = 0.0677; Fig 5, Table 2), but average within-species pairwise nucleotide diversity (*π*), determined from re-sequencing 10 males of the Guanapo population, is significantly higher on the X-chromosome (p < 0.001; Table 2).

**Table 2:** Characteristics of autosomes and X chromosome (LG12). Estimates are from 50 kb windows across each linkage group. Measurements are *d*_*N*_ (rate of nonsynonymous substitutions per nonsynonymous site), *d*_*S*_ (rate of synonymous substitutions per synonymous site) and the ratio of *d*_*N*_/*d*_*S*_ (mean estimates are approximated by *Σd*_*N*_*/Σd*_*S*_), *π* (nucleotide diversity within populations). Coverage was estimated using pooled resequencing data and is shown as average coverage per base. Statistical testing was carried out using non-parametrical Mann-Whitney *U* test (n.s., not significant, ***, p < 0.001).

Feature	Autosomes	LG12 (Chromosome X)	MW*U* Test
*d*_*N*_	0.0086	0.0085	n.s
*d*_*S*_	0.0579	0.0652	n.s
*d*_*N*_*/d*_*S*_	0.1855	0.1619	n.s
*π*	0.0025	0.0039	***
Coverage	110	110	n.s.
GC content	39.40%	39.04%	***
Genic	28.34%	27.86%	n.s.
Repeat content	7.62%	7.54%	n.s.

Numerous lines of evidence point to both X- and Y-chromosomes harboring genes for male size and color (see Introduction), including a previous QTL study that used approximately 800 SNP markers [30]. We therefore explored the QTL regions in more detail. Of three markers on the proximal end of LG12 that explained variation in male size [30], we localized two, marker-30 and marker-61, on scaffold 10 at positions 6,071,651 and 6,634,572. This region (6.0–6.7 Mb), harbors two genes encoding growth-related proteins, epidermal growth factor-like protein 7 (*egf17*) [73], and growth arrest-specific protein 1-like (*GAS1*) [74], (S7 Table). From blast searches using known candidate genes as queries as well as alignments of all LG12 scaffolds to public databases we found another six candidates for growth across this chromosome (S7 Table). Tripathi and colleagues [30] also found markers on LG12 explaining color variation [30]; we could locate three of them, marker_691, marker_423, and marker_210, on scaffold 13 at positions 4,566,793, 5,622,647 and 7,057,973. Within this region (4.6–7.1 Mb) we identified one possible coloration gene candidate, *mlana* [75]. Additional reciprocal blast searches revealed four other coloration gene candidates distributed throughout LG12: Solute Carrier Family 45 Member A2 (*slc45a2*, *aim1*) [76], Superkiller Viralicidic Activity 2-Like 2 (*skiv2l2*) [77], prepromelanin concentrating hormone (*pro-MCH-like*) [78], and Sepiapterin Reductase (*spra*) [79] (S7 Table).

Tripathi and colleagues [30] had mapped the sex-determining locus to the distal-most position of LG12, a region possibly not included in our female genome assembly. Of note, three genes related to sex differentiation in fishes (*parm1* [80], 5-hydroxytryptamine receptor 1A-beta-like [81], and *gadd45gamma* [82]) and one to sex-linked behavior (*5ht receptor 1a* [81]) were found within 120 kb of each other on one of the short distal scaffolds (scaffold 185; S7 Table).

Reads from the 10 Guanapo wild-caught male individuals (details about these individuals are given in the next subsection and in Methods section) that could not be aligned to the female reference genome were assembled separately. This resulted in 1,462 contigs between 1 and 7.5 kb length (mean 1.6 kb), summing up to 2.3 Mb of sequence that potentially represent male-specific regions from the Y-like differentiated part of the sex chromosomes. These sequences include 72 protein-coding genes with and 34 without putative function, each of which covers at least 40% of a homolog in the *nr* database (S8 Table).

### Population resequencing

To investigate sequence diversity within the Guanapo population, the origin of the strain used for genome assembly, we resequenced 10 wild-caught males, with a mean coverage of 12x per individual (range 8.5x to 14.0x). Mapping of the male reads to the female reference genome identified almost 5 million single nucleotide polymorphisms (SNPs). About 80% of SNPs were detected in at least two alleles (S9 Table). On average, slightly more than 2 million SNPs were identified per individual and the average ratio of heterozygous to homozygous SNPs was 1.76 (±0.16, median 1.74; S10 Table). 10% of SNPs were located in coding regions, with 173,485 nonsynonymous substitutions, and 2,520 nonsense changes relative to the reference (S11 Table). Average pairwise nucleotide diversity (*π*) was 0.0025 (±0.0013, median 0.0024) with a very homogeneous distribution across the entire genome, and limited within-chromosome variation along LG2, LG5, LG8, LG16 and LG18 (Fig 2). Inspecting the distribution of the uppermost 1% of *π* windows estimates (*π* > 0.009) further showed no spatial clustering along the chromosomes (Kolmogorov-Smirnov test D = 0.2145, p = 0.2324).

Nucleotide composition (measured as GC content averaged across 50 kb windows) was weakly negatively correlated with *π* (Spearman’s ⍴ = -0.0216, p = 0.0299). Inspecting this correlation further showed that for low and average (35%-41%) GC content the correlation was negative, whereas for higher GC content (>41%) the correlation showed a positive trend (S2 Fig). We found no correlation between *π* and number of genic sites per window (⍴ = -0.0146, p = 0.1419), but there was a small negative correlation between *π* and repeat content (⍴ = -0.0295, p = 0.0030).

**Fig 6:**
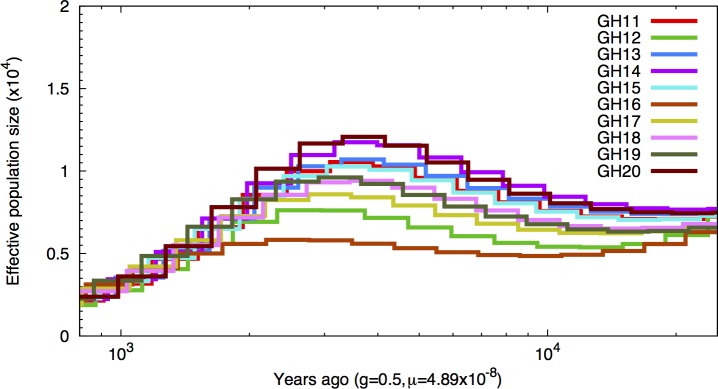
Inference of population size changes over time. PSMC results for the high-predation population for each individual. Each color represents a single individual. Time scale on the x-axis is calculated assuming a mutation rate of 4.89x10^-8^ bp^-1^ generation^-1^ and a generation time of 0.5 years.

**Fig 7:**
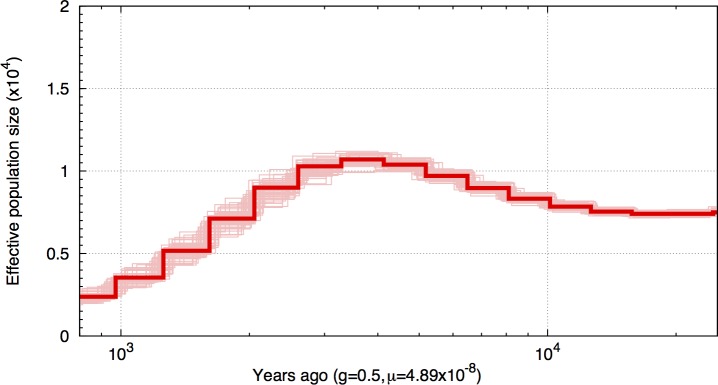
Bootstrapped inference of population size change over time. Plots of bootstrapped PSMC results for single individuals representing the high-predation population (GH13). The solid red line depicts the average estimate, the light red lines the 50 bootstrap results. Time scale on the x-axis is calculated assuming a mutation rate of 4.89x10^-8^ bp^-1^ generation^-1^ and a generation time of 0.5 years.

The historical demography of the resequenced individuals was examined using a coalescent-based Hidden Markov model [62] for each of the resequenced individuals. The ancestral effective population (*N*_*e*_) size was estimated to be highest about three to four thousand years ago (*N*_*e*_ ~10,000) and to have declined since then at a fairly constant rate (Fig 6). To verify the observed pattern, we bootstrapped the analysis. The bootstrap results (50 bootstraps) confirmed the demographic history of the high-predation Guanapo guppy population (shown only for individual GH13, see Fig 7).

## Discussion

Here, we present a high-quality assembled reference genome for the evolutionary and ecological model system, the Trinidadian guppy. The total assembly size is approximately 732 Mb, close to the predicted genome size of 740 to 900 Mb derived from flow cytometry and Feulgen stain densitometry [66]. The assembly is highly contiguous compared to other published teleost genomes (S12 Table), with half the assembly represented by 43 scaffolds that are at least 5.3 Mb long. We achieved a high quality assembly, even though inbreeding was relatively limited. For comparison, over 100 generations of inbreeding preceded efforts to generate a whole genome assembly for *Xiphophorus maculatus*, another species from the same family as the guppy [83]. Using a high-density linkage-map, we oriented 219 scaffolds (94% of the assembled genome) along 23 chromosomes. We found a high amount of synteny with medaka and stickleback, and verified that LG2 is the result of a fusion between two ancestral chromosomes [30], further confirming the high degree of karyotypes stability in percomorph fishes (e.g. [83]). We also fully assembled the mitochondrial genome (Supplementary Text, S3 Fig), which can be used to better understand the phylogenetic relationships among distantly related fishes [84]. Together, the female reference genome greatly enhances the molecular resources that have been developed for this system [45, 85–87]. The new reference assembly has already helped to determine the molecular basis of color mutations [86] and it has informed comparisons of natural and experimental populations [88].

The guppy reference genome will help to uncover the genetic basis of adaptive phenotypes in the guppy. A previous QTL analysis provided preliminary evidence that several loci involved in male size and coloration are located on the sex chromosome, with the sex-determining locus situated at the most distal end [30]. After anchoring these markers in the assembled genome, we searched for candidate genes in their vicinity and identified several genes with known functions in pigmentation, growth, and sex-determination in other taxa (see S7 Table for references). Likewise, several research groups interested in the relationship between female mate choice and male-specific coloration attempted to characterize the visual pigment (opsin) gene complement of the guppy, with special focus on the multiple and diversified LWS genes, but results varied across studies (e.g., depending on whether guppy genomic DNA or eye RNA was screened) [70, 89, 90]. Our results validate the combined results of these previous studies, identifying four LWS opsins, two RH2 opsins, two SWS2 opsins, one SWS1 opsin, and one RH1 opsin. Comparison of this Guanapo guppy genome assembly to the previous genomic Cumana guppy BAC sequencing results agree in LWS copy number and arrangement, including a retrotransposed LWS4 within the gepherin gene. Watson *et al*. [68] have previously compared genomic structure of all LWS genes between the Cumana guppy and *Xiphophorus helleri* by genomic BAC sequencing and concluded that the arrangement of the LWS cluster as well as the LWS-4 retrotransposition event occurred before the split between *Xiphophorus* and *Poecilia* clades [68, 69].

We are particularly interested in the evolution of the sex chromosomes, LG12, because of its known variation underlying traits important in local adaptation such as male color and size. Sex chromosomes are predicted to evolve differently compared to autosomes because of their differences in transmission and ploidy, and the resulting differences in effective population size [91]. We have, however, not found evidence for an elevated rate of X-chromosome evolution in the guppy. This may be due to the fact that the majority of the assembled chromosome is pseudoautosomal and freely recombines with the Y-chromosome while the diversified differentiated distal region might still be missing in the assembly. Moreover, sex determination is a rapidly evolving trait in fishes including the Poeciliids, and estimates of evolutionary rates using medaka and platy as outgroups are almost certainly dominated by time periods when LG12 sequences were autosomal. Within-species nucleotide diversity was higher on the X chromosome than on the autosomes, which suggests that molecular evolutionary differences between the guppy sex chromosomes and autosomes do become apparent at shorter time-scales.

Since we cannot tell whether a female counterpart of the differentiated region of the Y-chromosome exists in females, we separately assembled reads from ten male samples, and identified contigs that did not map to the female reference assembly. Although we assembled one thousand such contigs, none contained obvious candidates for sex determination, pigmentation, or growth. These male-only contigs were short in length (the longest just over 7 kb) and harbored many truncated open reading frames, which may be due to incomplete assembly, or because they had undergone pseudogenization. The absence of recombination in Y-chromosomes is predicted to reduce natural selection in this region and, in turn, increase the rate of pseudogenization, gene-loss, and repeat expansion [92, 93]. A third alternative is that sequences are mostly shared between X and Y, with male-specific sequences interspersed between shared sequences, rather than long blocks of male-specific sequences.

We have already exploited the reference assembly to investigate genetic diversity in the Guanapo river, a high predation locality in Northwest Trinidad that was the source population for our reference strains. Diversity was fairly homogeneous across the genome and was not strongly correlated with other genomic features such as GC or genic content. Coverage could be another potential confounding factor with respect to pairwise nucleotide diversity. We found that with higher coverage slightly fewer alternative alleles were called (⍴ = -0.1147, p < 0.001, S4 Fig) but the correlation was not linear and did not significantly change the proportion of alternative alleles called between regions of different coverage. While the correlation appeared positive for regions of lower coverage (70x-130x), it was negative for regions of higher coverage (130x-300x).

Using a pairwise sequential Markovian coalescent model [62] that uses local density of heterozygous sites in individual diploids, we estimated that our source population had a large current effective population size (~2,500). This was comparable to other estimates of effective population sizes of a high predation population estimated from the same river (~1,300 [16]) and other lowland high predation populations (~2,000–14,000 [88], ~700–4,200 [16]). Estimates of *N*_*e*_ grew with increasing coalescence age, which is expected if the population is well connected via gene flow to a larger meta-population. This extensive genetic variation found in lowland populations, could be a major contributor to the rapid and repeatable adaptation of colonizers to novel predation regimes seen in further upstream locations [19, 20].

Our reference assembly presents an important step in providing a much-needed resource for the study of evolutionary genetics in the guppy. Future studies can make use of our reference assembly and explore the many aspects of guppy biology that make it a model system in understanding evolutionary biology and ecology, including life history evolution, maternal provisioning, and invasion success. The limitations of our short-read based reference assembly, however, also highlight that new genome sequencing and assembly approaches are needed to reveal the complete sequence of the sex chromosomes in this species.

## Supporting Information

S1 Fig Whole genome alignment between guppy and stickleback. **(A)** Circos plot showing the syntenic relationship between guppy linkage groups 1–23 and UN for unassigned scaffolds and stickleback chromosomes I-XXI. **(B)** Circos plot highlighting alignments between guppy LG2 (left) and medaka chromosomes. **(C)** Circos plot for alignments between selected regions from guppy (LG2 and LG12) and stickleback. Each line represents an alignment block of at least 500 bp.
(PDF)

S2 Fig Nucleotide composition (GC-content) in correlation with average nucleotide diversity (*π*). GC content and *π* were estimated from 50 kb windows. The orange line represents the LOWESS regression; dashed lines denote mean GC content and mean *π*, respectively.
(PDF)

S3 Fig Guppy mitochondrial genome. Circos plot of annotation of mitochondrial genome. The outermost circle denotes genes and rRNAs/tRNAs transcribed from the leading strand, and the second outermost circle from the lagging strand. The innermost circle represents the GC content per every 5 bp; the darker lines are, the higher the GC content.
(PDF)

S4 Fig Coverage in correlation with proportion of alleles called. Mean coverage was estimated from 50 kb windows. The number of reference and non-reference sites per SNP was counted and the proportion per 50 kb window was estimated for the reference allele (Proportion Ref Allele) and for the non-reference allele (Proportion Alt Allele). The orange line represents the LOWESS regression; the vertical dashed line denotes mean coverage.
(PDF)

S1 Table Overview of sequencing data used for assembling the female guppy genome. Raw data denotes sequencing yield after *phiX* removal and filtered data denotes sequence data after removing PCR duplicates. PE = Paired-end library, MP = Mate pair library, FM = Fosmid library.
(PDF)

S2 Table Overview of sequencing data used for population resequencing and population. Sequencing yield for high predation population individuals. Column ‘Reads mapping to reference’ refers to the percentage of reads mapping to the female genome. The column ‘Uncovered in Mb’ refers to the uncovered sequences in Mb after mapping the paired-end genomic libraries to the reference genome.
(PDF)

S3 Table Rhodopsin and opsin genes found in the genome assembly. (PDF)

S4 Table Predictions of small RNA loci using Infernal and predicted tRNAs and potential pseudogenes coding tRNAs using tRNA-scan. (PDF)

S5 Table Repeat content of the female guppy genome as identified by RepeatMasker. In total, 156,122,771 bp (~21.3%) of the assembly were classified as repeats.
(PDF)

S6 Table Linkage group sizes after anchoring scaffolds. Linkage group Un contains all unanchored scaffolds.
(PDF)

S7 Table Protein coding genes related to (A) growth, (B) pigment pattern and (C) sex differentiation on LG12.
(PDF)

S8 Table Best hit protein-coding genes retrieved for male specific sequences. (XLSX)

S9 Table Frequency of alleles different from the reference in resequencing populations (single nucleotide polymorphisms only). (PDF)

S10 Table Number of single nucleotide polymorphisms per individual and ratio of heterozygous to homozygous SNPs. (PDF)

S11 Table Single nucleotide polymorphisms by type and region for resequencing data inferred by SnpEff. Note SNPs can be counted in two or more categories.
(PDF)

S12 Table Selected published fish genome assemblies. Comparison of 10 different published fish genomes and the guppy genome. Chromosomes refer to whether the assembly is available as chromosomes from GenBank.
(PDF)

S13 Table Protein coding genes with putative functions in pigment pattern development, vision, growth and sex differentiation. (XLSX)

S1 File Supplementary Text. Additional information about procedures and analyses related to the guppy genome project.
(PDF)
