# Supplementary figures and images for: The Genome of the Trinidadian Guppy, Poecilia reticulata, and Variation in the Guanapo Population
Source: PLoS One. 2016 Dec 29;11(12):e0169087. doi: 10.1371/journal.pone.0169087 (PMC5199103; doi:10.1371/journal.pone.0169087)

**A**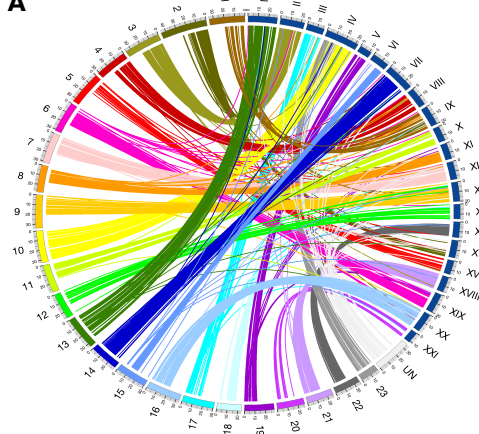**B**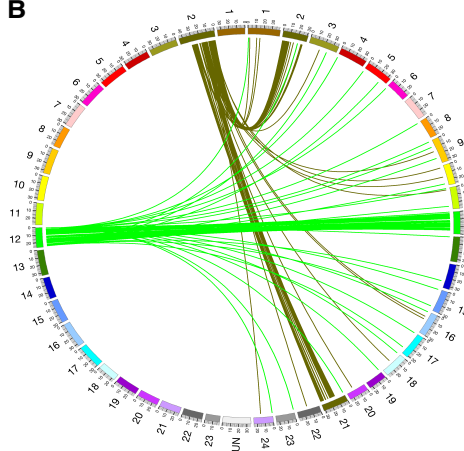**C**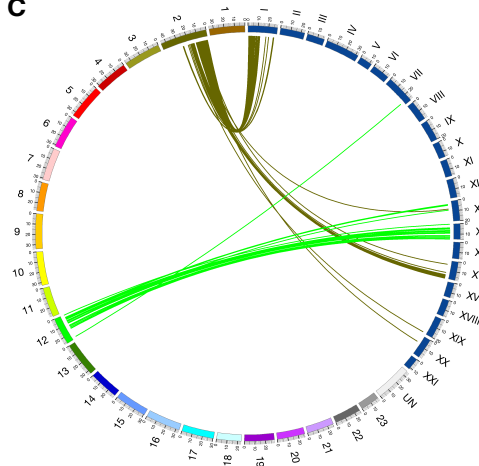

Supplement: S1 Fig — (A) Circos plot showing the syntenic relationship between guppy linkage groups 1–23 and UN for unassigned scaffolds and stickleback chromosomes I-XXI. (B) Circos plot highlighting alignments between guppy LG2 (left) and medaka chromosomes. (C) Circos plot for alignments between selected regions from guppy (LG2 and LG12) and stickleback. Each line represents an alignment block of at least 500 bp. (PDF) [file pone.0169087.s001.pdf]

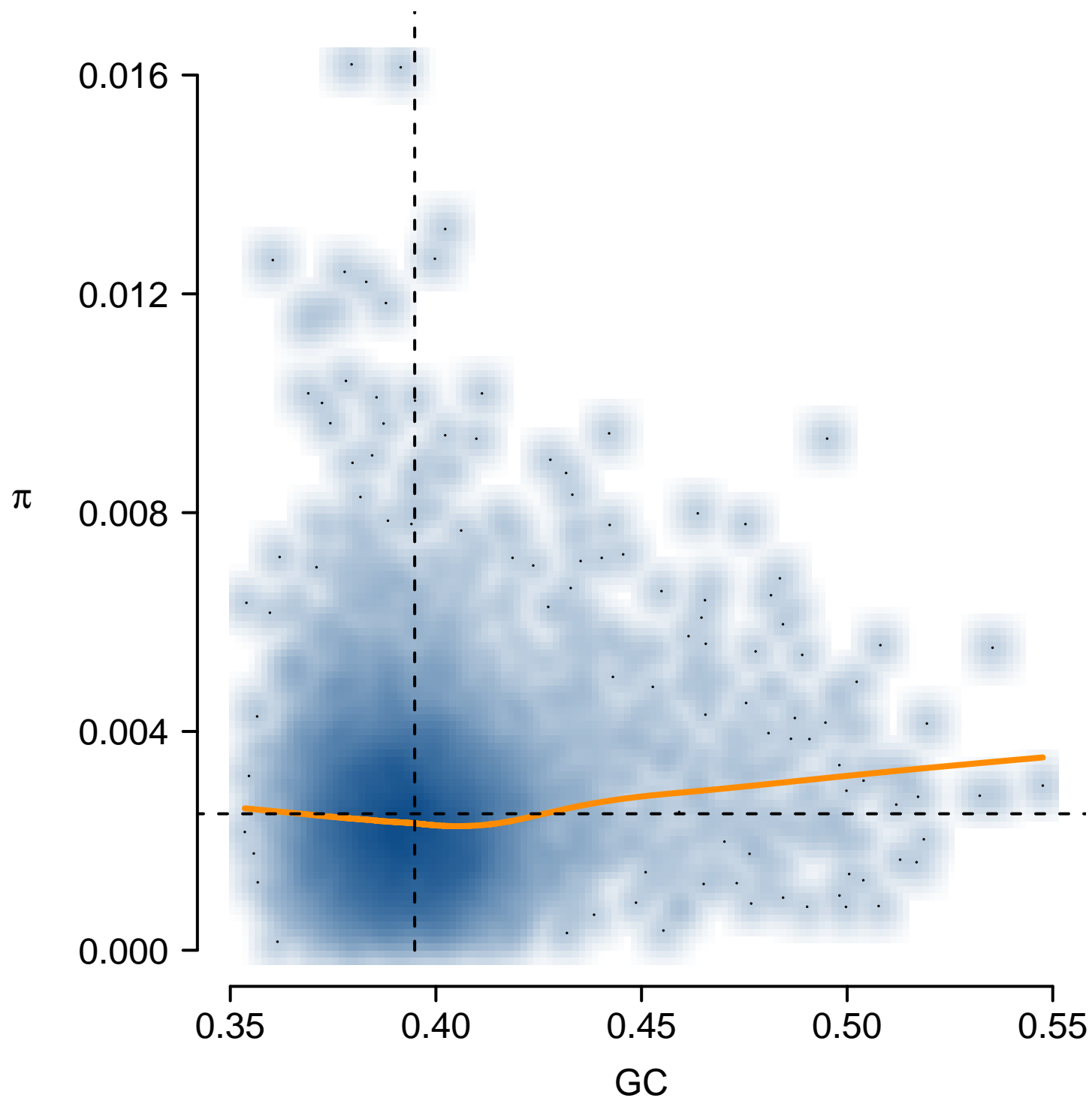

Supplement: S2 Fig — GC content and π were estimated from 50 kb windows. The orange line represents the LOWESS regression; dashed lines denote mean GC content and mean π, respectively. (PDF) [file pone.0169087.s002.pdf]

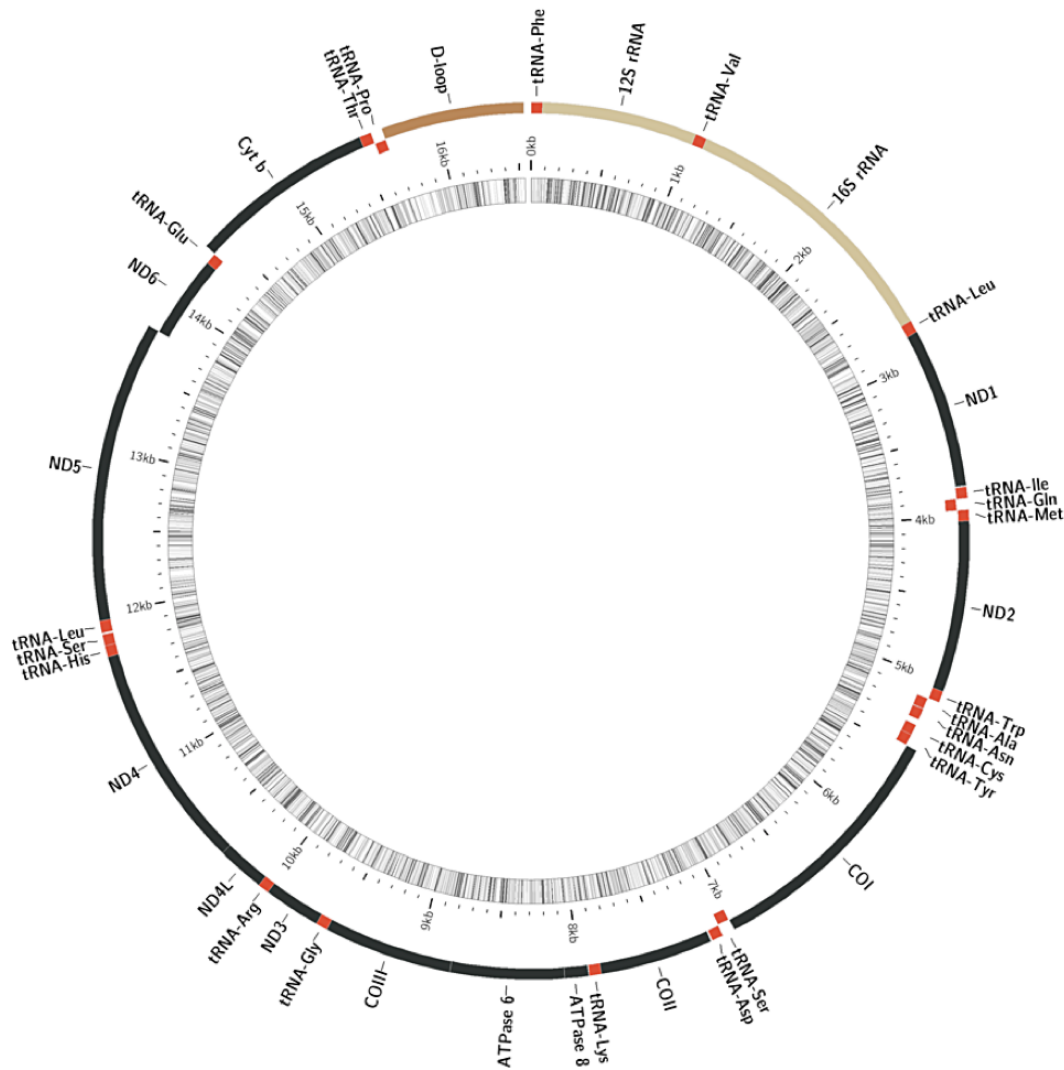

Supplement: S3 Fig — Circos plot of annotation of mitochondrial genome. The outermost circle denotes genes and rRNAs/tRNAs transcribed from the leading strand, and the second outermost circle from the lagging strand. The innermost circle represents the GC content per every 5 bp; the darker lines are, the higher the GC content. (PDF) [file pone.0169087.s003.pdf]

Proportion Ref Allele - Proportion Alt Allele

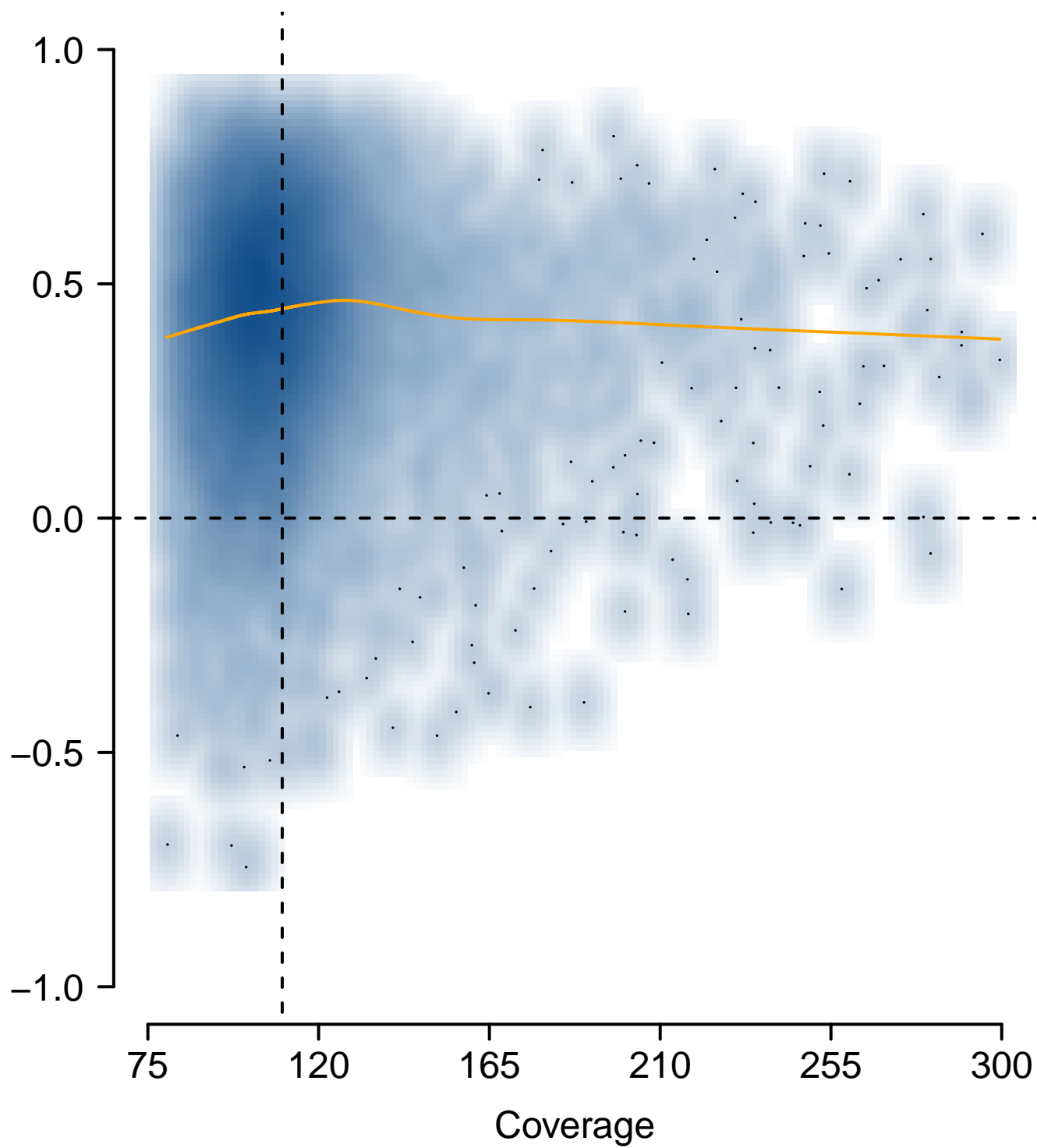

Supplement: S4 Fig — Mean coverage was estimated from 50 kb windows. The number of reference and non-reference sites per SNP was counted and the proportion per 50 kb window was estimated for the reference allele (Proportion Ref Allele) and for the non-reference allele (Proportion Alt Allele). The orange line represents the LOWESS regression; the vertical dashed line denotes mean coverage. (PDF) [file pone.0169087.s004.pdf]
