# Supplementary material for: The Genome of the Trinidadian Guppy, Poecilia reticulata, and Variation in the Guanapo Population
Source: PLoS One. 2016 Dec 29;11(12):e0169087. doi: 10.1371/journal.pone.0169087 (PMC5199103; doi:10.1371/journal.pone.0169087)
Supplement: S1 Table — Raw data denotes sequencing yield after phiX removal and filtered data denotes sequence data after removing PCR duplicates. PE = Paired-end library, MP = Mate pair library, FM = Fosmid library. (PDF) [file pone.0169087.s005.pdf]

S1 Table. Overview of sequencing data used for assembling the female guppy genome.

Raw data denotes sequencing yield after *phiX* removal and filtered data denotes sequence data after removing PCR duplicates. PE = Paired-end library, MP = Mate pair library, FM = Fosmid library.

ID	Library insert size	Library Type	Raw Read length	Raw data in Gb	Filtered data in Gb	SRA Accession
1	240	PE	151	11.7	11.7	SRR1171023
2	240	PE	136	11.9	11.9	SRR1171023
3	270	PE	151	12.5	12.5	SRR1171024
4	270	PE	151	12.2	11.2	SRR1171024
5	270	PE	151	13.0	13.0	SRR1171024
6	460	PE	151	11.5	11.5	SRR1171025
7	460	PE	151	11.2	11.2	SRR1171025
8	460	PE	151	5.2	5.2	SRR1171025
9	3000	MP	151	10.0	6.3	SRR1171308
10	3000	MP	151	10.7	9.5	SRR1171308
11	3000	MP	151	10.9	2.9	SRR1171308
12	5500	MP	151	12.8	6.4	SRR1171309
13	8000	MP	151	8.8	9.5	SRR1171310
14	13000	MP	151	11.4	3.7	SRR1171311
15	14000	MP	151	8.5	1.9	SRR1171312
16	20000	MP	151	13.9	6.2	SRR1171313
17	20000	MP	151	10.8	4.4	SRR1171313
18	40000	MP	151	11.9	2.8	SRR1171314
19	45-60000	MP	151	11.7	2.9	SRR1171315
20	40000	FM	101	14.4	3.0	SRR1171306
