# Supplementary material for: The Genome of the Trinidadian Guppy, Poecilia reticulata, and Variation in the Guanapo Population
Source: PLoS One. 2016 Dec 29;11(12):e0169087. doi: 10.1371/journal.pone.0169087 (PMC5199103; doi:10.1371/journal.pone.0169087)
Supplement: S2 Table — Sequencing yield for high predation population individuals. Column ‘Reads mapping to reference’ refers to the percentage of reads mapping to the female genome. The column ‘Uncovered in Mb’ refers to the uncovered sequences in Mb after mapping the paired-end genomic libraries to the reference genome. (PDF) [file pone.0169087.s006.pdf]

S2 Table. Overview of sequencing data used for population resequencing and population.

Sequencing yield for high predation population individuals. Column 'Reads mapping to reference' refers to the percentage of reads mapping to the female genome. The column 'Uncovered in Mb' refers to the uncovered sequences in Mb after mapping the paired-end genomic libraries to the reference genome.

ID	Raw data in Gb	Filtered data in Gb	Coverage per site	Reads mapping reference (in %)	Uncovered in Mb	SRA Accession
GH11	11.4	9.9	13.2	91.4%	82.7	SRR1171699
GH12	8.4	7.0	9.6	92.0%	83.6	SRR1171701
GH13	12.4	10.7	14.0	90.2%	87.9	SRR1171702
GH14	11.8	10.2	13.6	92.2%	74.8	SRR1171703
GH15	11.7	10.0	13.0	89.5%	76.2	SRR1171704
GH16	9.7	8.3	8.5	68.9%	87.7	SRR1171705
GH17	9.6	8.2	11.0	91.3%	83.8	SRR1171707
GH18	10.6	9.0	12.0	91.3%	79.0	SRR1171708
GH19	10.6	8.7	11.7	91.7%	75.4	SRR1171709
GH20	11.8	10.4	13.8	91.4%	76.4	SRR1171710
