# Supplementary material for: The Genome of the Trinidadian Guppy, Poecilia reticulata, and Variation in the Guanapo Population
Source: PLoS One. 2016 Dec 29;11(12):e0169087. doi: 10.1371/journal.pone.0169087 (PMC5199103; doi:10.1371/journal.pone.0169087)
Supplement: S3 Table — (PDF) [file pone.0169087.s007.pdf]

**S3 Table. Rhodopsin and opsin genes found in the genome assembly.**

Note that the cluster of SWS2A, SWS2B, LWS-1, LWS-2, and LWS-3 on the Cumaná BAC 35E7 (HM540108) has been annotated in reverse order relative to chromosome [3].

| Guppy Opsin | LG | NCBI Gene ID | Position on scaffold (mRNA) | GenBank Accession | Reference |
|---|---|---|---|---|---|
| RH2_1 | 5 | XP_008406984.1 | scaffold_31:5601938-5604011 | DQ234859.1 | [1] |
| RH2_2 | 5 | XP_008406985.1 | scaffold_31:5616145-5620938 | DQ234858.1 | [1] |
| LWS-1 (A180) | 5 | XP_008408728.1 | scaffold_43:4295595-4297764 | HQ260675.1; HM540108.1 | [2, 3] |
| LWS-2 (P180) | 5 | XP_008408794.1 | scaffold_43:4280373-4290244 | AB748956.1; HM540108.1 | [3, 4] |
| LWS-3 (S180) | 5 | XP_008408727.1 | scaffold_43:4274724-4276879 | HQ260677.1; HM540108.1 | [2, 3] |
| SWS2B | 5 | XP_008408729.1 | scaffold_43:4306172-4308530 | DQ234860.1; HM540108.1 | [1, 3] |
| SWS2A | 5 | XP_008408730.1 | scaffold_43:4310261-4313196 | JF303638.1; HM540108.1 | [2, 3] |
| LWS-4 (S180r) | 21 | XP_008395693.1 | scaffold_19:2104249-2111012 | HQ260678.1; HM540107.1 | [2, 3] |
| SWS1 | UN | XP_008399945.1 | scaffold_125:1382757-1384348 | DQ234861.1 | [1] |
| RH-1 (Rhodopsin) | 7 | XP_008412214.1 | scaffold_1:6207362-6208968 | DQ912023.1 | [1] |


1. Hoffmann M, Tripathi N, Henz SR, Lindholm AK, Weigel D, Breden F, et al. Opsin gene duplication and diversification in the guppy, a model for sexual selection. Proc Biol Sci. 2007;274(1606):33-42. doi: 10.1098/rspb.2006.3707. PubMed PMID: 17015333; PubMed Central PMCID: PMCPMC1679887.
2. Laver CR, Taylor JS. RT-qPCR reveals opsin gene upregulation associated with age and sex in guppies (Poecilia reticulata) - a species with color-based sexual selection and 11 visual-opsin genes. BMC Evol Biol. 2011;11:81. Epub 2011/03/31. PubMed PMID: 21447186; PubMed Central PMCID: PMC3078887.
3. Watson CT, Gray SM, Hoffmann M, Lubieniecki KP, Joy JB, Sandkam BA, et al. Gene duplication and divergence of long wavelength-sensitive opsin genes in the guppy, Poecilia reticulata. J Mol Evol. 2011;72(2):240-52. Epub 2010/12/21. doi: 10.1007/s00239-010-9426-z. PubMed PMID: 21170644.
4. Tezuka A, Kasagi S, van Oosterhout C, McMullan M, Iwasaki WM, Kasai D, et al. Divergent selection for opsin gene variation in guppy (Poecilia reticulata) populations of Trinidad and Tobago. Heredity (Edinb). 2014;113(5):381-9. doi: 10.1038/hdy.2014.35. PubMed PMID: 24690753; PubMed Central PMCID: PMCPMC4220713.
