# Supplementary material for: The Genome of the Trinidadian Guppy, Poecilia reticulata, and Variation in the Guanapo Population
Source: PLoS One. 2016 Dec 29;11(12):e0169087. doi: 10.1371/journal.pone.0169087 (PMC5199103; doi:10.1371/journal.pone.0169087)
Supplement: S4 Table — (PDF) [file pone.0169087.s008.pdf]

S4 Table. Predictions of small RNA loci using INFERNAL and predicted tRNAs and potential pseudogenes coding tRNAs using TRNA-SCAN.

Program	Class	Number
Infernal	5S rRNA	15
	miRNA	707
	RNase P	4
	rRNA	30
	snoRNA	160
	snRNA	88
	tRNA	419
tRNA-scan	tRNAs	439
	Selenocysteine tRNAs (TCA)	3
	Possible suppressor tRNAs (CTA,TTA)	1
	undetermined tRNAs	65
	Predicted pseudogenes coding tRNAs	4186
