# Supplementary material for: The Genome of the Trinidadian Guppy, Poecilia reticulata, and Variation in the Guanapo Population
Source: PLoS One. 2016 Dec 29;11(12):e0169087. doi: 10.1371/journal.pone.0169087 (PMC5199103; doi:10.1371/journal.pone.0169087)
Supplement: S5 Table — In total, 156,122,771 bp (~21.3%) of the assembly were classified as repeats. (PDF) [file pone.0169087.s009.pdf]

S5 Table. Repeat content of the female guppy genome as identified by REPEATMASKER.

In total, 156,122,771 bp (~21.34%) of the assembly were classified as repeats.

Class		number of elements	length occupied (bp)	percentage of sequence
SINEs		26,112	4,330,869	0.59
	ALUs	0	0	0.00
	MIRs	2,413	350,008	0.05
LINEs		65,412	14,641,359	2.00
	LINE1	775	166,359	0.02
	LINE2	32,210	6,824,858	0.93
	L3/CR1	0	0	0.00
LTR elements		12,329	3,573,918	0.49
	ERVL	0	0	0.00
	ERVL-MaLRs	0	0	0.00
	ERV_classI	249	133,863	0.02
	ERV_classII	471	36,309	0.00
DNA elements		329,048	63,347,538	8.66
	hAT-Charlie	32,074	8,651,932	1.18
	TcMar-Tigger	766	150,224	0.02
Unclassified		332,799	58,681,468	8.02
Total interspersed repeats			144,575,152	19.76
Small RNA		4,420	622,426	0.09
Satellites		2,879	637,506	0.09
Simple repeats		119,216	6,549,682	0.90
Low complexity		121,946	4,917,112	0.67
