# Supplementary material for: The Genome of the Trinidadian Guppy, Poecilia reticulata, and Variation in the Guanapo Population
Source: PLoS One. 2016 Dec 29;11(12):e0169087. doi: 10.1371/journal.pone.0169087 (PMC5199103; doi:10.1371/journal.pone.0169087)
Supplement: S6 Table — Linkage group Un contains all unanchored scaffolds. (PDF) [file pone.0169087.s010.pdf]

S6 Table. Linkage group sizes after anchoring scaffolds.

Linkage group Un contains all unanchored scaffolds.

Linkage group	Length in Mb
1	34.11
2	46.29
3	35.26
4	31.50
5	33.91
6	31.53
7	31.41
8	27.94
9	34.12
10	32.82
11	28.87
12	26.44
13	33.52
14	28.34
15	30.64
16	33.20
17	30.79
18	22.03
19	28.47
20	26.38
21	25.77
22	25.25
23	18.08
Un	34.94
