# Supplementary material for: The Genome of the Trinidadian Guppy, Poecilia reticulata, and Variation in the Guanapo Population
Source: PLoS One. 2016 Dec 29;11(12):e0169087. doi: 10.1371/journal.pone.0169087 (PMC5199103; doi:10.1371/journal.pone.0169087)
Supplement: S7 Table — Protein coding genes related to (A) growth, (B) pigment pattern and (C) sex differentiation on LG12. (PDF) [file pone.0169087.s011.pdf]

S7 Table. Protein coding genes related to (A) growth, (B) pigment pattern and (C) sex differentiation found on LG12.

A. Genes related to growth

Scaffold	NCBI mRNA	NCBI protein	Predicted protein	Reference
10	XM_008423749.1	XP_008421971.1	BDNF/NT-3 growth factors receptor-like	[1]
10	XM_008423561.1	XP_008421783.1	epidermal growth factor-like protein 7	[2]
10	XM_008423514.1	XP_008421736.1	growth arrest-specific protein 1-like (GAS1)	[3]
10	XM_008423783.1	XP_008422005.1	growth/differentiation factor 7-like (GSDF)	[4]
10	XM_008423158.1	XP_008421380.1	Basic fibroblast growth factor receptor 1A-like (GHR1a)	[5]
13	XM_008424055.1	XP_008422277.1	fibroblast growth factor 10-like	[6]
188	XM_008424590.1	XP_008422812.1	teratocarcinoma-derived growth factor 1-like (TDGF1, one-eyed pinhead)	[7]
185	XM_008424758.1	XP_008422980.1	growth hormone receptor-like (GHR2)	[5]

B. Genes related to pigment pattern formation

10	XM_008423759.1	XP_008421981.1	superkiller viralicidic activity 2-like 2 (skiv2l2)	[8]
10	XM_008423772.1	XP_008421994.1	SLC45A2 (aim1)	[9]
80	XM_008424603.1	XP_008422825.1	prepromelanin concentrating hormone (pro-MCH-like)	[10]
231	XM_008424833.1	XP_008423055.1	sepiapterin reductase (spra)	[11]

C. Genes related to sex differentiation

10	XM_008423078.1	XP_008421300.1	nuclear receptor subfamily 5 group A member 1b (NR5A1b, SF1)	[12]
13	XM_008423879.1	XP_008422101.1	progestin and adipoQ receptor family member 3-like	[13]
13	XM_008424014.1	XP_008422236.1	spermatid perinuclear RNA-binding protein	[14]
13	XM_008424087.1	XP_008422309.1	stAR-related lipid transfer protein 7	[15]

13	XM_008424101.1	XP_008422323.1	germ cell-less homolog 1	[16]
13	XM_008424116.1	XP_008422338.1	oxysterol-binding protein 2-like	[17]
13	XM_008424492.1	XP_008422714.1	prolactin receptor	[18]
13	XM_008424151.1	XP_008422373.1	prolactin-releasing peptide receptor-like	[19]
188	XM_008424583.1	XP_008422805.1	spindlin1-like (spin4, 1 of 4)	[20]
188	XM_008424565.1	XP_008422787.1	one cut domain family member 2	[21]
185	XM_008424751.1	XP_008422973.1	prostate androgen-regulated mucin-like protein 1 homolog (parm1)	[22]
185	XM_008424748.1	XP_008422970.1	5-hydroxytryptamine receptor 1A-beta-like	[23]
185	XM_008424735.1	XP_008422957.1	growth arrest and DNA damage-inducible protein GADD45 gamma-like	[24]

1. Germana A, Laura R, Montalbano G, Guerrera MC, Amato V, et al. (2010) Expression of brain-derived neurotrophic factor and TrkB in the lateral line system of zebrafish during development. Cell Mol Neurobiol 30: 787-793.
2. Shen MM, Schier AF (2000) The EGF-CFC gene family in vertebrate development. Trends Genet 16: 303-309.
3. Airaksinen MS, Holm L, Hatinen T (2006) Evolution of the GDNF family ligands and receptors. Brain Behav Evol 68: 181-190.
4. Myosho T, Otake H, Masuyama H, Matsuda M, Kuroki Y, et al. (2012) Tracing the emergence of a novel sex-determining gene in medaka, Oryzias luzonensis. Genetics 191: 163-170.
5. Ellens ER, Kittilson JD, Hall JA, Sower SA, Sheridan MA (2013) Evolutionary origin and divergence of the growth hormone receptor family: insight from studies on sea lamprey. Gen Comp Endocrinol 192: 222-236.
6. Liu F, Pogoda HM, Pearson CA, Ohyama K, Lohr H, et al. (2013) Direct and indirect roles of Fgf3 and Fgf10 in innervation and vascularisation of the vertebrate hypothalamic neurohypophysis. Development 140: 1111-1122.
7. Skinner MK, Schmidt M, Savenkova MI, Sadler-Riggleman I, Nilsson EE (2008) Regulation of granulosa and theca cell transcriptomes during ovarian antral follicle development. Mol Reprod Dev 75: 1457-1472.
8. Yang CT, Hindes AE, Hultman KA, Johnson SL (2007) Mutations in gfpt1 and skiv2l2 cause distinct stage-specific defects in larval melanocyte regeneration in zebrafish. PLoS Genet 3: e88.
9. Fukamachi S, Asakawa S, Wakamatsu Y, Shimizu N, Mitani H, et al. (2004) Conserved function of medaka pink-eyed dilution in melanin synthesis and its divergent transcriptional regulation in gonads among vertebrates. Genetics 168: 1519-1527.
10. Kawauchi H, Baker BI (2004) Melanin-concentrating hormone signaling systems in fish. Peptides 25: 1577-1584.
11. Negishi S, Fujimoto K, Katoh S (2003) Localization of sepiapterin reductase in pigment cells of Oryzias latipes. Pigment Cell Res 16: 501-503.
12. Kuo MW, Postlethwait J, Lee WC, Lou SW, Chan WK, et al. (2005) Gene duplication, gene loss and evolution of expression domains in the vertebrate nuclear receptor NR5A (Ftz-F1) family. Biochem J 389: 19-26.

13. Thomas P, Pang Y (2012) Membrane progesterone receptors: evidence for neuroprotective, neurosteroid signaling and neuroendocrine functions in neuronal cells. Neuroendocrinology 96: 162-171.
14. Meikar O, Vagin VV, Chalmel F, Sostar K, Lardenois A, et al. (2014) An atlas of chromatoid body components. RNA 20: 483-495.
15. Miller WL (2013) Steroid hormone synthesis in mitochondria. Mol Cell Endocrinol 379: 62-73.
16. Cinalli RM, Lehmann R (2013) A spindle-independent cleavage pathway controls germ cell formation in Drosophila. Nat Cell Biol 15: 839-845.
17. Ramachandra RK, Lankford SE, Weber GM, Rexroad CE, 3rd, Yao J (2007) Identification of OORP-T, a novel oocyte-specific gene encoding a protein with a conserved oxysterol binding protein domain in rainbow trout. Mol Reprod Dev 74: 502-511.
18. Whittington CM, Wilson AB (2013) The role of prolactin in fish reproduction. Gen Comp Endocrinol 191: 123-136.
19. Watanabe S, Kaneko T (2010) Prolactin-releasing peptide receptor expressed in the pituitary in Mozambique tilapia Oreochromis mossambicus: an aspect of prolactin regulatory mechanisms. Gen Comp Endocrinol 167: 27-34.
20. Chew TG, Peaston A, Lim AK, Lorthongpanich C, Knowles BB, et al. (2013) A tudor domain protein SPINDLIN1 interacts with the mRNA-binding protein SERBP1 and is involved in mouse oocyte meiotic resumption. PLoS One 8: e69764.
21. Gladden JM, Meyer BJ (2007) A ONECUT homeodomain protein communicates X chromosome dose to specify Caenorhabditis elegans sexual fate by repressing a sex switch gene. Genetics 177: 1621-1637.
22. Park JY, Jang H, Curry TE, Sakamoto A, Jo M (2013) Prostate androgen-regulated mucin-like protein 1: a novel regulator of progesterone metabolism. Mol Endocrinol 27: 1871-1886.
23. Loveland JL, Uy N, Maruska KP, Carpenter RE, Fernald RD (2014) Social status differences regulate the serotonergic system of a cichlid fish, Astatotilapia burtoni. J Exp Biol 217: 2680-2690.
24. Johnen H, Gonzalez-Silva L, Carramolino L, Flores JM, Torres M, et al. (2013) Gadd45g is essential for primary sex determination, male fertility and testis development. PLoS One 8: e58751.
