# Supplementary material for: The Genome of the Trinidadian Guppy, Poecilia reticulata, and Variation in the Guanapo Population
Source: PLoS One. 2016 Dec 29;11(12):e0169087. doi: 10.1371/journal.pone.0169087 (PMC5199103; doi:10.1371/journal.pone.0169087)
Supplement: S9 Table — (PDF) [file pone.0169087.s013.pdf]

S9 Table. Frequency of alleles different from the reference in resequencing populations (single nucleotide polymorphisms only).

Frequency of alleles	Number of changes compared to reference
1	944,963
2	642,701
3	481,457
4	424,684
5	360,200
6	322,395
7	265,579
8	241,341
9	205,397
10	200,745
11	150,375
12	133,398
13	112,790
14	100,176
15	85,856
16	75,149
17	60,858
18	57,140
19	46,520
20	54,298
SNPs total	4,966,022
