# Supplementary material for: The Genome of the Trinidadian Guppy, Poecilia reticulata, and Variation in the Guanapo Population
Source: PLoS One. 2016 Dec 29;11(12):e0169087. doi: 10.1371/journal.pone.0169087 (PMC5199103; doi:10.1371/journal.pone.0169087)
Supplement: S10 Table — (PDF) [file pone.0169087.s014.pdf]

S10 Table. Number of single nucleotide polymorphisms per individual and ratio of heterozygous to homozygous SNPs.

ID	No of SNPs	Ratio heterozygous to homozygous SNPs
GH11	2,172,270	1.77
GH12	1,937,937	1.46
GH13	2,117,353	1.68
GH14	2,243,913	1.89
GH15	2,225,037	1.94
GH16	2,089,162	1.62
GH17	2,125,450	1.70
GH18	2,164,406	1.71
GH19	2,258,495	1.88
GH20	2,237,011	1.97
