# Supplementary material for: The Genome of the Trinidadian Guppy, Poecilia reticulata, and Variation in the Guanapo Population
Source: PLoS One. 2016 Dec 29;11(12):e0169087. doi: 10.1371/journal.pone.0169087 (PMC5199103; doi:10.1371/journal.pone.0169087)
Supplement: S11 Table — Note SNPs can be counted in two or more categories. (PDF) [file pone.0169087.s015.pdf]

S11 Table. Single nucleotide polymorphisms by type and region for resequencing data inferred by SnpEff.

Note SNPs can be counted in two or more categories.

Category/Impact		Number of changes
Coding	Missense	173,485
	Nonsense	2,520
	Silent	247,674
Noncoding	Upstream 5'	1,676,362
	Downstream 3'	1,692,488
	Intergenic	1,821,797
