# Supplementary material for: The Genome of the Trinidadian Guppy, Poecilia reticulata, and Variation in the Guanapo Population
Source: PLoS One. 2016 Dec 29;11(12):e0169087. doi: 10.1371/journal.pone.0169087 (PMC5199103; doi:10.1371/journal.pone.0169087)
Supplement: S12 Table — Comparison of 10 different published fish genomes and the guppy genome. Chromosomes refer to whether the assembly is available as chromosomes from GenBank. (PDF) [file pone.0169087.s016.pdf]

S12 Table. Selected published fish genome assemblies.

Comparison of 10 different published fish genomes and the guppy genome. Chromosomes refer to whether the assembly is available as chromosomes from GENBANK.

Species	Organism name	Assembly size in Mb	Number Scaffolds	Scaffolds per Mb	L50 in Mb	Chromosomes	GenBank Assembly ID	Sequencing technology
Amazon molly	*Poecilia formosa*	749	3,985	5.32	1.57	no	GCA_000485575.1	Illumina
Atlantic cod	*Gadus morhua*	824	427,427	518.72	0.39	no	GCA_000231765.1	454
Burton's mouthbrooder	*Haplochromis burtoni*	831	8,001	9.63	1.19	no	GCA_000239415.1	Illumina
Coelacanth	*Latimeria chalumnae*	2,860	22,818	7.98	0.92	no	GCA_000225785.1	Illumina
Guppy	*Poecilia reticulata*	732	3,029	4.14	5.27	yes	GCA_000633615.2	Illumina
Mexican tetra	*Astyanax mexicanus*	1,191	10,735	9.01	1.78	no	GCA_000372685.1	Illumina
Nile tilapia	*Oreochromis niloticus*	928	5,901	6.36	2.80	no	GCA_000188235.1	Illumina
Northern pike	*Esox lucius*	878	5,688	6.48	0.70	yes	GCA_000721915.1	Illumina
Platyfish	*Xiphophorus maculatus*	730	20,632	28.26	1.30	no	GCA_000241075.1	454, ABI 3730
Spotted gar	*Lepisosteus oculatus*	946	2,106	2.23	6.93	yes	GCA_000242695.1	Illumina
Tongue sole	*Cynoglossus semilaevis*	470	31,181	66.34	0.51	yes	GCA_000523025.1	Illumina
