# Supplementary material for: The Genome of the Trinidadian Guppy, Poecilia reticulata, and Variation in the Guanapo Population
Source: PLoS One. 2016 Dec 29;11(12):e0169087. doi: 10.1371/journal.pone.0169087 (PMC5199103; doi:10.1371/journal.pone.0169087)
Supplement: S1 File — Additional information about procedures and analyses related to the guppy genome project. (PDF) [file pone.0169087.s018.pdf]

Supplementary Text

Genome assembly

Mitochondrial DNA assembly. To assemble the guppy mitochondrial DNA one paired-end library was selected (library ID 3). Due to the high difference in coverage between the genomic DNA and the mtDNA, a transcriptome *de novo* assembler (TRINITY version r2012-03-17 [1]) was chosen to assemble the data with default parameters.

The resulting assembly was screened for components (contigs) that might have originated from the mitochondrial DNA. The complete mtDNA from *Xiphophorus maculatus* (NC_011379.1) was downloaded from NCBI and blasted against the assembly (BLASTN version 2.2.21 [2]). Components with significant hits (e-value < 10^{-20}) were inspected and assembled to build the circular mtDNA.

The mtDNA (16,538 bp) was annotated using MITOANNOTATOR [3]. All 13 mitochondria genes, 22 tRNAs, 2 rRNAs, and the d-loop region were found in the guppy mtDNA (Figure S3). Sequence and annotations were submitted to GenBank (Accession KJ460033).

Genome size estimation. According to the ANIMAL GENOME SIZE DATABASE (www.genomesize.com, last accessed 2013-09-25), the C-value of the haploid guppy genome is between 0.77 and 1.00 pg, where 1 pg corresponds to 978 Mb [4], although genome size may differ among guppy populations [5]. We used all paired-end libraries to estimate genome size, using a *k-mer* approach. *K-mers* were counted using JELLYFISH version 1.1.6 [6], resulting in an estimate 779.8 Mb for the female genome. Since our assembly is 731.6 Mb, it would cover 93.8% of the estimated size. Missing might be regions of extreme GC-content [7] or collapsed repeat regions [8].

Final assembly screening. During submission of the genome assembly, screening for small sequence stretches (<200 bp) flanked by Ns of length greater than 10 bp was done by NCBI. In total, 23 short stretches of sequences were removed, with a total of 2,614 bp. Additionally, scaffolds with missing sequence information (Ns) at the beginning or the end of a scaffold were shortened (3 scaffolds, 5,522 bp in total).

Assembly validation

Markers from a recent genetic linkage map (N=5,493, see below section 'Physical assembly') were blasted (BLASTN version 2.2.27+, e-value < 10^{-20}) against the genome assembly and 94.6% of the markers had a hit against the genome (296 markers without a hit).

The ESTs, contigs, and linkage map markers not found in our assembly may be misassembled contigs or contaminants, or strain-specific transcripts, male specific transcripts, or representing regions not present in the assembly.

To evaluate the completeness of the female guppy genome using sequences conserved outside teleost fishes, a set of core eukaryotic genes (CEGs) were mapped using Core Eukaryotic Genes Mapping Approach (CEGMA; [9]). CEGMA uses a set of conserved protein families to construct alignments between the proteins and draft assemblies. The genome assembly was uploaded to the CEGMA website

(http://korflab.ucdavis.edu/Datasets/cegma/submit.html, last access 2014-03-21) and evaluation was run with default parameters for vertebrate species. The assembled genome contains 223 full-length CEGS out of 248 highly conserved CEGs (89.9%). Using the less conserved gene set, 433 full-length CEGs out of 458 CEGs (94.5%) were found in the guppy assembly.

Physical assembly

A genetic linkage map with ~800 markers has been published [10]. We updated this map using 184 F_2 progeny, their parents and grandparents of the previously used cross 157 [10]. Markers for genotyping were retrieved following a RAD-seq approach as described [11, 12]. 5,493 markers were used to build a comprehensive linkage map using Joinmap4 [13]. This updated genetic map was used to order and orientate scaffolds along the 23 guppy linkage groups as described below.

Markers were aligned against the assembled genome (BLASTN, e-value < 10^{-20}) and 5.161 (90.2%) markers could be placed with unique hits. We were able to place 284 scaffolds (696.67 Mb, 95.2%) onto linkage groups. Out of these, 219 scaffolds (686.42 Mb, 93.8% of the assembly) could be orientated along linkage groups. Details are given in Figure 3 and Table S6.

Thirty-three discrepancies were found between the linkage map and the assembly. The majority (23) occurred at the beginning or the end of scaffolds. We did not correct these errors because there may be differences in chromosomal composition, *e.g.* rearrangements, between different guppy populations [5]. The linkage map was created using a cross between individuals from the Quare and the Cumaná populations. Both populations are quite divergent from the Guanapo population [14], which was used for genome sequencing. The remaining 10 discrepancies were single markers that aligned to a linkage group not predicted by the genetic map. The total number of discrepancies was used to calculate the overall assembly error rate (ε)

$$\varepsilon = \frac{\varphi}{\sigma}$$

with φ being the number of incorrectly placed markers and σ the number of scaffolds containing a marker. We calculated an assembly error rate of 0.035 (3.5%) for the assembly. This number is an upper bound for ε, because local rearrangements between different guppy strains might lead to an increase in ε.

Annotations

Protein coding genes. For *ab initio* prediction, the AUGUSTUS web-server [15, 16] was used. First, parameters for the prediction process were trained with 4,434 guppy EST sequences randomly chosen from NCBI. Next, *ab initio* prediction was performed resulting in a total of 33,527 gene models.

For the RNA-seq approach, gene models were assembled using genome-guided TRINITY (http://trinityrnaseq.sourceforge.net/genome_guided_trinity.html, last accessed 2012-11-30). RNA-seq reads from whole embryos and adult tissues [17] were first normalized using *in silico* read normalization with TRINITY [18]. Normalized reads from each dataset were individually assembled for reconstruction of sample-specific transcriptomes. Briefly, reads were first mapped to the reference genome using GSNAP v2012-07-20 [19]. The

mapped reads were partitioned into read-covered regions of the genome and reads in each partition were *de novo* assembled with TRINITY. All transcripts from each assembly were combined and clustered using CD-HIT-EST version 4.6.1 [20] with default parameters, resulting in 360,506 clusters. To further reduce the number of clusters, we first applied USEARCH version 6.0.307 [21] with cluster_fast and 80% identity option on the resulting sequences. In a second step, we applied CD-HIT-EST a second time (sequence identity threshold 0.8) to get a final set of clustered transcripts.

The set of clustered transcripts was cleaned with SEQCLEAN (PASA version r2012-06-25) to prepare it as input for PASA [22]. PASA was run with a maximum intron length of 100,000 bp. Support for terminal exons was calculated with the *retrieve_terminal_CDS_exons.pl* script delivered with PASA.

For the orthology-based approach, protein sequences from zebrafish (*Danio rerio*), stickleback (*Gasterosteus aculeatus*), cod (*Gadus morhua*), and medaka (*Oryzias latipes*) were downloaded from ENSEMBL (version 70) [23]. For each gene, we extracted the longest amino acid sequence (total = 86,176) and clustered the sequences using CD-HIT with sequence identity threshold set to 0.7. This resulted in 48,803 protein sequence clusters. Next, the clusters were blasted (TBLASTP version 2.2.27+, maximum intron length 100,000 bp, e-value < 10^{-5}) against the draft genome sequence. All protein clusters with confident hits (45,526 sequences) were aligned against the guppy draft assembly using EXONERATE version 2.2.0 [24] with the protein2genome model, with at least a 60% match to the genome and maximum intron length of 200,000 bp. This approach resulted in 10,627 orthologous gene models.

Gene models from all three approaches were combined to build the guppy reference gene set. We used EVIDENCEMODELER version r2012-06-25 [25] and used different weights on the prediction methods (weights for the different methods: *ab initio* 4, protein 5, transcript 10). EVIDENCEMODELER was run on 1 Mb genome segments with 100 kb overlap to reduce computational burden. The resulting reference gene set contained 31,902 protein-coding sequences. The predicted gene models are available from the Max-Planck-Institute's website (ftp://ftp.tuebingen.mpg.de/ebio/publication_data/akunstner/; gff - pret.gene_models.20130422.gff, cds - pret.gene_models.20130422.cds.fa, proteins - pret.gene_models.20130422.proteins.fa).

RNA-seq data from several tissues [17] were mapped to the gene models using BOWTIE2 version 2.1.0 [26]. Approximately 51% of the RNA-seq reads could be mapped to the genome, covering the majority of the predicted gene models (98.4%, 31,390).

Gene set annotation. All sequences from the reference gene set were uploaded to the ORTHOMCL Release V5 website [27]. For approximately 80% of the sequences (25,047), we were able to retrieve an assignment to an orthologous group. Additionally, 689 sequences were annotated as within-species paralogs.

As a second source of annotation, PROTEINORTHO version 4.26 [28] was run (parameters: e-value < 10^{-5}, minimum coverage of best blast hit 0.4, minimum similarity for additional hits 0.8) using protein sequences from anole lizard (*Anolis carolinensis*, ENSEMBL 71), chicken (*Gallus gallus*, ENSEMBL 70), cod (*Gadus morhua*, ENSEMBL 70), fugu (*Takifugu rubripes*, ENSEMBL 71), human (*Homo sapiens*, ENSEMBL 71), medaka (*Oryzias latipes*, ENSEMBL 70), mouse (*Mus musculus*, ENSEMBL 71), platyfish (*Xiphophorus maculatus*, ENSEMBL 71), stickleback (*Gasterosteus aculeatus*, ENSEMBL 70), tilapia (*Oreochromis niloticus*, ENSEMBL 71), and zebrafish (*Danio rerio*, ENSEMBL 70). All data was downloaded from ENSEMBL and for each species we kept only the longest protein

coding sequence for each ortholog. Thereby we retrieved annotations for 17,317 guppy protein sequences. In total, annotations for 25,277 (79.2%) guppy sequences could be retrieved from either ORTHOMCL or PROTEINORTHO. Approximately one fifth of the guppy genes (6,625) remain without annotation.

Gene ontology annotations were found using BLAST2GO version 2.6.6 [29]. First, genes were matched against the NCBI non-redundant database (*nr*) using BLASTX (maximum number of hits retained = 5, e-value < 10^{-3}). The resulting matches were then mapped to GO terms (e-value hit filter = 10^{-6}, annotation cutoff = 55, GO weight = 5) (databases were accessed 2013-05-22).
Approximately 35.3% (11,247) of gene models could be assigned at least one GO term (8,276 biological process; 9,224 molecular function; 7,001 cellular component).

Comparison EVM gene set *vs.* NCBI gene set. To evaluate the completeness of each gene set, three-way alignments between guppy, medaka and stickleback were created. Coding sequences from medaka and stickleback were downloaded from BIOMART (ENSEMBL 70) and orthology relationship to guppy (1:1:1 orthologs) was identified using PROTEINORTHO version 5.07 (parameter settings: minimum similarity for additional hits 0.8, BLASTP+). For the EVM gene set, 7,488 1:1:1 orthologs could be identified whereas 9,870 could be retrieved for the NCBI gene set. Additionally, we have looked at the number of missing genes. Missing genes were defined as ortholog sequences between medaka and stickleback that could not be found in guppy. In the EVM gene set 5,346 genes were missing, in the NCBI gene set 3,778. Furthermore, mean and median length of the gene models was significantly longer in the NCBI gene set compared to the EVM predictions (Mann-Whitney *U* test, $p < 0.001$, NCBI mean/median gene length: 2,172 bp/1.557 bp; EVM mean/median: 1,060 bp/729 bp). We conclude that the NCBI gene set is more comprehensive compared to the EVM gene set and used the NCBI gene predictions for all further analyses.

Supplemental References

1. Grabherr MG, Haas BJ, Yassour M, Levin JZ, Thompson DA, Amit I, et al. Full-length transcriptome assembly from RNA-Seq data without a reference genome. Nature Biotechnology. 2011;29(7):644-52. doi: 10.1038/nbt.1883.
2. Altschul SF, Madden TL, Schäffer AA, Zhang J, Zhang Z, Miller W, et al. Gapped BLAST and PSI-BLAST: a new generation of protein database search programs. Nucleic Acids Res. 1997;25(17):3389-402.
3. Iwasaki W, Fukunaga T, Isagozawa R, Yamada K, Maeda Y, Satoh TP, et al. MitoFish and MitoAnnotator: A Mitochondrial Genome Database of Fish with an Accurate and Automatic Annotation Pipeline. Mol Biol Evol. 2013;30(11):2531-40. doi: 10.1093/molbev/mst141.
4. Gregory TR, Nicol JA, Tamm H, Kullman B, Kullman K, Leitch IJ, et al. Eukaryotic genome size databases. Nucl Acids Res. 2007;35(Database issue):D332-8. doi: 10.1093/nar/gkl828. PubMed PMID: 17090588; PubMed Central PMCID: PMCPMC1669731.
5. Nanda I, Schories S, Tripathi N, Dreyer C, Haaf T, Schmid M, et al. Sex chromosome polymorphism in guppies. Chromosoma. 2014. doi: 10.1007/s00412-014-0455-z. PubMed PMID: 24676866.
6. Marcais G, Kingsford C. A fast, lock-free approach for efficient parallel counting of occurrences of k-mers. Bioinformatics (Oxford, England). 2011;27(6):764-70. doi: 10.1093/bioinformatics/btr011. PubMed PMID: 21217122; PubMed Central PMCID: PMCPMC3051319.
7. Ellegren H, Smeds L, Burri R, Olason PI, Backström N, Kawakami T, et al. The genomic landscape of species divergence in Ficedula flycatchers. Nature. 2012;491(7426):756-60. doi: 10.1038/nature11584.
8. Miller J, Koren S, Sutton G. Assembly algorithms for next-generation sequencing data. Genomics. 2010;95(6):315-27. doi: 10.1016/j.ygeno.2010.03.001.
9. Parra G, Bradnam K, Korf I. CEGMA: a pipeline to accurately annotate core genes in eukaryotic genomes. Bioinformatics (Oxford, England). 2007;23(9):1061-7. doi: 10.1093/bioinformatics/btm071. PubMed PMID: 17332020.
10. Tripathi N, Hoffmann M, Willing EM, Lanz C, Weigel D, Dreyer C. Genetic linkage map of the guppy, Poecilia reticulata, and quantitative trait loci analysis of male size and colour variation. Proceedings of the Royal Society B: Biological Sciences. 2009;276(1665):2195-208. doi: 10.1098/rspb.2008.1930. PubMed PMID: 19324769; PubMed Central PMCID: PMCPMC2677598.
11. Baird N, Etter P, Atwood T, Currey M, Shiver A, Lewis Z, et al. Rapid SNP Discovery and Genetic Mapping Using Sequenced RAD Markers. 2008;3(10):e3376. doi: 10.1371/journal.pone.0003376.
12. Willing EM, Hoffmann M, Klein JD, Weigel D, Dreyer C. Paired-end RAD-seq for de novo assembly and marker design without available reference. Bioinformatics (Oxford, England). 2011;27(16):2187-93. doi: 10.1093/bioinformatics/btr346. PubMed PMID: 21712251.
13. van Ooijen JW. Multipoint maximum likelihood mapping in a full-sib family of an outbreeding species. Genet Res (Camb). 2011;93(5):343-9. doi: 10.1017/S0016672311000279. PubMed PMID: 21878144.
14. Willing E-M, Bentzen P, van Oosterhout C, Hoffmann M, Cable J, Breden F, et al. Genome-wide single nucleotide polymorphisms reveal population history and adaptive divergence in wild guppies. Molecular Ecology. 2010;19(5):968-84. doi: 10.1111/j.1365-294X.2010.04528.x. PubMed PMID: 20149094.
15. Hoff K, Stanke M. trainAUGUSTUS-A Webserver Application for Parameter Training and Gene Prediction in Eukaryotes. International Plant & Animal XX Conference 2012, USA. 2012. PubMed PMID: DD906A75-8AD8-4983-9C44-A49C6495227A.
16. Hoff KJ, Stanke M. WebAUGUSTUS--a web service for training AUGUSTUS and predicting genes in eukaryotes. Nucl Acids Res. 2013;41(Web Server issue):W123-8. doi: 10.1093/nar/gkt418. PubMed PMID: 23700307; PubMed Central PMCID: PMCPMC3692069.
17. Sharma E, Künstner A, Fraser BA, Zipprich G, Kottler VA, Henz SR, et al. Transcriptome assemblies for studying sex-biased gene expression in the guppy, Poecilia reticulata. BMC Genomics. 2014;15:400. doi: 10.1186/1471-2164-15-400. PubMed PMID: 24886435; PubMed Central PMCID: PMC4059875.
18. Haas B, Papanicolaou A, Yassour M, Grabherr M, Blood P, Bowden J, et al. De novo transcript sequence reconstruction from RNA-seq using the Trinity platform for reference generation and analysis. Nature Protocols. 2013;8(8):1494-512. doi: 10.1038/nprot.2013.084.
19. Wu TD, Nacu S. Fast and SNP-tolerant detection of complex variants and splicing in short reads. Bioinformatics (Oxford, England). 2010;26(7):873-81. doi: 10.1093/bioinformatics/btq057. PubMed PMID: 20147302; PubMed Central PMCID: PMCPMC2844994.
20. Fu L, Niu B, Zhu Z, Wu S, Li W. CD-HIT: accelerated for clustering the next-generation sequencing data. Bioinformatics (Oxford, England). 2012;28(23):3150-2. doi: 10.1093/bioinformatics/bts565.
21. Edgar RC. Search and clustering orders of magnitude faster than BLAST. Bioinformatics (Oxford, England). 2010;26(19):2460-1. doi: 10.1093/bioinformatics/btq461. PubMed PMID: 20709691.

22. Haas BJ, Zeng Q, Pearson MD, Cuomo CA, Wortman JR. Approaches to Fungal Genome Annotation. Mycology. 2011;2(3):118-41. doi: 10.1080/21501203.2011.606851. PubMed PMID: 22059117; PubMed Central PMCID: PMCPMC3207268.
23. Flicek P, Ahmed I, Amode MR, Barrell D, Beal K, Brent S, et al. Ensembl 2013. Nucl Acids Res. 2013;41(Database issue):D48-55. doi: 10.1093/nar/gks1236. PubMed PMID: 23203987; PubMed Central PMCID: PMCPMC3531136.
24. Slater GS, Birney E. Automated generation of heuristics for biological sequence comparison. BMC Bioinformatics. 2005;6. doi: 10.1186/1471-2105-6-31. PubMed PMID: 15713233; PubMed Central PMCID: PMCPMC553969.
25. Haas BJ, Salzberg SL, Zhu W, Pertea M, Allen JE, Orvis J, et al. Automated eukaryotic gene structure annotation using EVidenceModeler and the Program to Assemble Spliced Alignments. Genome Biol. 2008;9(1):R7. doi: 10.1186/gb-2008-9-1-r7.
26. Langmead B, Salzberg SL. Fast gapped-read alignment with Bowtie 2. Nature Methods. 2012;9(4):357-9. doi: 10.1038/nmeth.1923. PubMed PMID: 22388286.
27. Chen F, Mackey AJ, Vermunt JK, Roos DS. Assessing performance of orthology detection strategies applied to eukaryotic genomes. PLoS ONE. 2007;2. doi: 10.1371/journal.pone.0000383.
28. Lechner M, Findeiß S, Steiner L, Marz M, Stadler PF, Prohaska SJ. Proteinortho: Detection of (Co-)orthologs in large-scale analysis. BMC Bioinformatics. 2011;12(1):124. doi: 10.1073/pnas.0708855104. PubMed PMID: 21526987.
29. Conesa A, Götz S. Blast2GO: A Comprehensive Suite for Functional Analysis in Plant Genomics. International Journal of Plant Genomics. 2008;2008(3):1-12. doi: 10.1093/nar/gkl197. PubMed PMID: 18483572.
